# Estimation of internal displacement in Ukraine from satellite-based car detections

**DOI:** 10.1038/s41598-024-80035-8

**Published:** 2024-12-30

**Authors:** Marie-Christine Rufener, Ferda Ofli, Masoomali Fatehkia, Ingmar Weber

**Affiliations:** 1 Qatar Computing Research Institute, Hamad Bin Khalifa University, Doha, Qatar; 2 Computer Science Department, Saarland University, Saarbrücken, Germany

**Keywords:** Car Detection, Satellite Imagery, Convolutional Neural Network, Crisis Response, Migration, Societal Computing, Computer science, Psychology and behaviour

## Abstract

Estimating the numbers and whereabouts of internally displaced people (IDP) is paramount to providing targeted humanitarian assistance. In conflict settings like the ongoing Russia-Ukraine war, on-the-ground data collection is nevertheless often inadequate to provide accurate and timely information. Satellite imagery may sidestep some of these challenges and enhance our understanding of the IDP dynamics. Our study thus aimed to evaluate whether internal displacement patterns can be estimated from changes in car counts using multi-temporal satellite imagery. We collected over 1000 very-high-resolution images across Ukrainian cities between 2019 and 2022, to which we applied a state-of-the-art computer vision model to detect and count cars. These counts were then linked to population data to predict displacements through ratio or non-linear models. Our findings suggest a clear East-to-West movement of cars in the first months following the war’s onset. Despite data sparsity hindered fine-grained evaluation, we distinguished a clear positive and non-linear trend between the number of people and cars in most cities, which further allowed to predict the sub-national people dynamics. While our approach is resource-saving and innovative, satellite imagery and computer vision models present some shortcomings that could mask detailed IDPs dynamics. We conclude by discussing these limitations and outline future research opportunities.

**Supplementary Information:**

The online version contains supplementary material available at 10.1038/s41598-024-80035-8.

## Main

Millions of civilians are uprooted each year from their places of residence due to conflicts, human rights violations, and natural disasters^1^. The number of forcibly displaced people has more than doubled over the past decade, reaching a record high of 100 million in 2022^2^. Internally displaced people (IDPs), i.e., people forced to leave their homes but who remain within their country’s border, accounted for more than 70% of the recorded movements, marking a 20% increase compared to the previous year^3^. According to the latest report of the Internal Displacement Monitoring Centre (iDMC), the escalating number of conflicts and violence in the past year pushed the figure to an unprecedented 28.3 million displacements worldwide^3^. Such alarming numbers raise international concerns, as IDPs are amongst the most vulnerable people and often in need of humanitarian assistance to ensure their safety, as well as access to medical care, services, and food^1^.

While there have been wide-ranging international efforts to measure the scale of *cross-border* population flows, estimation of *within-country* migration flows is particularly prone to inaccuracies, lack of timely updates, and lack of disaggregation, e.g., by age^4,5^. This also applies in the context of the ongoing Russia-Ukraine War, which triggered the largest humanitarian crisis in Europe since World War II. As of October 2024, over 40 million border crossings from Ukraine have been registered since the start of the war (24 February 2022), in addition to 6.7 million Ukrainians who sought refuge in neighbouring countries and another 3.5 million within the borders of their country^6,7^.

The volatile situation of the conflict has resulted in pendular migration not only between Ukraine and adjacent countries but also within the country itself. Although public authorities have a good understanding of the population flow to and from receiving countries, less is known about the Ukrainians’ displacement pattern at the sub-national level^8^. This is a crucial limitation, as Ukraine currently lacks intermediary institutions that could ensure periodic assessments throughout the country.

To date, most information on IDPs is collected by humanitarian organizations through either phone- or field-based surveys^9,10^. In a conflict or disaster, however, these traditional data sources often fail to provide accurate and timely information, especially during the acute phase of the displacement crisis^5,9^. In armed conflicts, on-the-ground enumerators can be exposed to life-threatening risks, or have their access hindered by either infrastructural damages or inaccessibility to more remote areas^11^. As a consequence, most countries rely on a patchwork of IDP estimates that arise from various independent assessments, each of which is collected for different purposes that ultimately yield conflicting IDP estimates^9^.

In light of such limitations, the use of non-traditional data for monitoring mobility patterns has gained momentum among both the scientific and the humanitarian community due to their untapped potential to sidestep some of the current data limitations^9,12,13^. A substantial body of research has explored the potential use of anonymized call detail records (CDR) from mobile phone operators to monitor mobility patterns^14,15^. For example, studies have shown how CDR can track the spread of communicable diseases, such as the work by Wesolowski et al.^16,17^ on malaria, and Oliver et al.^18^ on COVID-19. Additionally, CDR data has been useful to measure communication patterns within particular communities such as the Syrian refugees in Turkey^19^, as well as to analyze internal displacement trends as exposed by Shibuya et al.^20^ in the context of the Russia-Ukraine War.

While CDR data is undoubtedly useful, procuring access is a major hurdle and needs to be negotiated for each telecom operator individually. Moreover, there are difficulties in quickly transferring a methodology from one country to another as different telecom operators might apply different processing and aggregation methods, and might fall under different national regulations. To address these concerns, a growing body of literature has been exploring digital traces from social media platforms, which are more easily accessible and persistent across countries [e.g., Refs.^8,10,21–23^]. Others, in turn, have been relying on satellite imagery to estimate population dynamics through either refugee settlement detection^12^ or analysis of nightlights data^24,25^.

Building upon these studies, we advocate that satellite imagery bears a fertile, and yet largely under-explored, ground for studying internal population displacements. News channels all around the world reported the fleeing of thousands of Ukrainians by means of personal or shared vehicles during the first weeks following the full-scale Russian invasion. Countless numbers of vehicles stretching dozens of kilometers have been recorded at the major checkpoints on the Western border, capturing the evasion of Ukrainians from the Eastern to the Western regions [e.g., Refs.^26–28^].

**Fig. 1:**
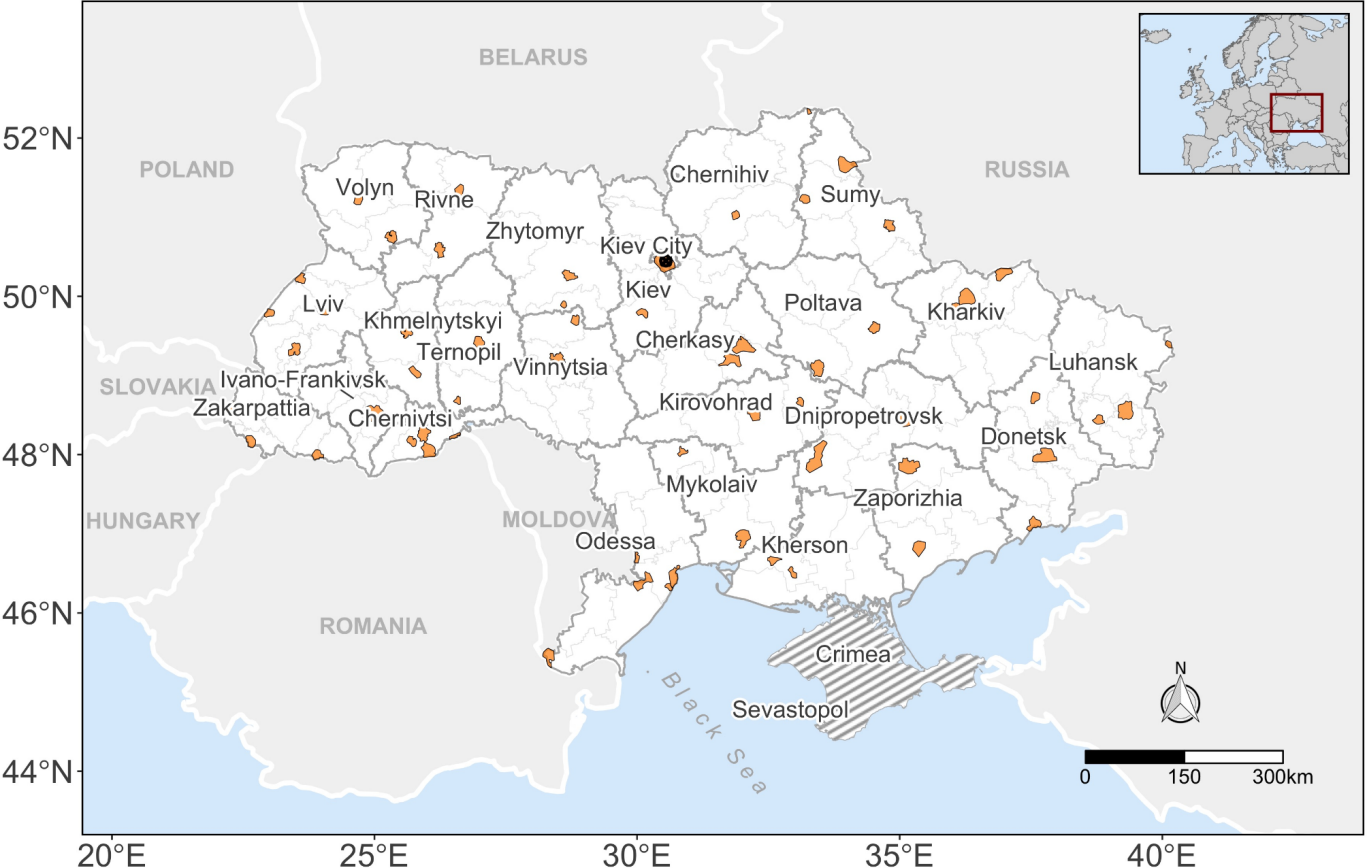
Map of the study region highlighting the selected areas of interest (AOI) in orange within each Oblast. An “oblast” in Ukraine is the main type of first-level administrative division, equivalent in hierarchy, though not in absolute size, to states in the US context. The gray-dashed area depicts the occupied territories of Crimea and Sevastopol, both excluded from the current study. This map was created using R software v.4.3.

This vehicle-facilitated displacement behavior raises the question of whether internal displacement patterns can be estimated from the spatial-temporal changes in car counts obtained from satellite imagery. To pursue this research question, we collected a total of 1009 very-high-resolution satellite images between 2019 and 2022, spanning 61 cities across Ukraine (Fig. 1; see section Material & Methods for more details about data collection and processing). Of these images, only 534 images remained for analysis after undergoing our data post-processing pipeline, with most cities remaining with less than 10 images throughout the time series (see Supplementary Fig. S1). Furthermore, the monthly data availability was generally sporadic for all cities, with several temporal gaps along the time series (i.e., months without any data) (see Supplementary Fig. S2). However, data coverage was much better during the months succeeding the start of the war than during the pre-conflict period, especially for cities heavily involved in the conflict (e.g., Kharkiv, Donetsk, Mariupol, Odessa, and Ivano-Frankivsk; see Supplementary Fig. S2). This likely reflects operational adjustments by the satellite operator in response to the demand for images from the conflict areas.

**Fig. 2:**
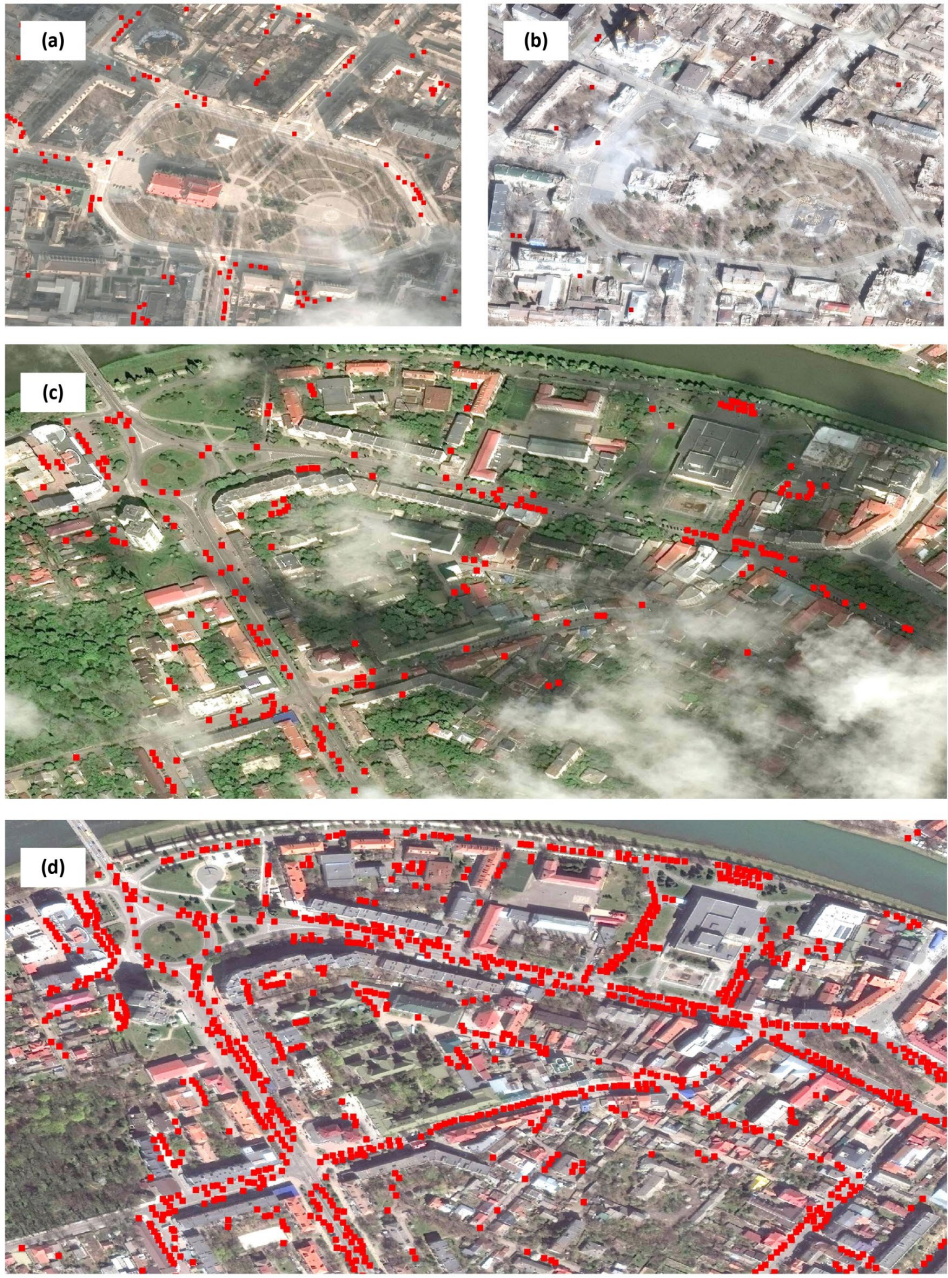
Satellite images showing the effect of war on the number of cars circulating in two distinct Ukrainian cities before and during the war. Panels (**a**) and (**b**) depict a snapshot of Mariupol before (12.02.2021) and during (29.03.2022) the war, respectively, highlighting the region around the Donetsk Academic Regional Drama Theatre that was heavily bombarded on 16th March. Panels (**c**) and (**d**) show close-up shots of the area around the Transcarpathian Regional Clinical Hospital of A. Novak before (30.04.2019) and during (14.04.2022) the war, respectively, located in the city of Uzhhorod. While Mariupol presented a massive drop in the number of cars in the first month following the start of the war, Uzhhorod depicted the opposite trend. Satellite images © 2019–2024 Maxar Technologies.

Despite the data shortages, visual inspection of these images revealed some clear pre-war vs. wartime patterns in the number of cars across multiple cities. For example, Mariupol, one of the most heavily affected cities by the war, suffered a massive drop in the number of cars during the first month succeeding the war when compared with the same month and region prior to the COVID-19 pandemic (Fig. 2a, b). The opposite trend was observed for the city of Uzhhorod on the Slovakian border, with a substantial increase in the number of vehicles during the months succeeding the outbreak of the war when contrasted to the pre-war period (Fig. 2c, d).

These two examples illustrate the apparent connection between the number of cars and people, as the car dynamics seemingly follow the general East-West migration pattern that has been previously reported for Ukraine^8,10^. On this basis, the current study proposes a novel methodology to estimate IDPs, using the ongoing Russia-Ukraine War as a motivating case study. By using a computer vision model in combination with a robust statistical analysis, we modelled the population-car relationship for multiple cities and estimated the sub-national people displacement whenever applicable.

## Results

In the following two sections, we first describe our more basic and measurement-centric analysis of (raw) car counts (see section “Spatio-temporal car dynamics”). Subsequently, we describe a more advanced and modelling-centric analysis that uses the changes in car counts to estimate changes in *population* counts (see section “Inferring IDPs from cars”).

### Spatio-temporal car dynamics

**Fig. 3:**
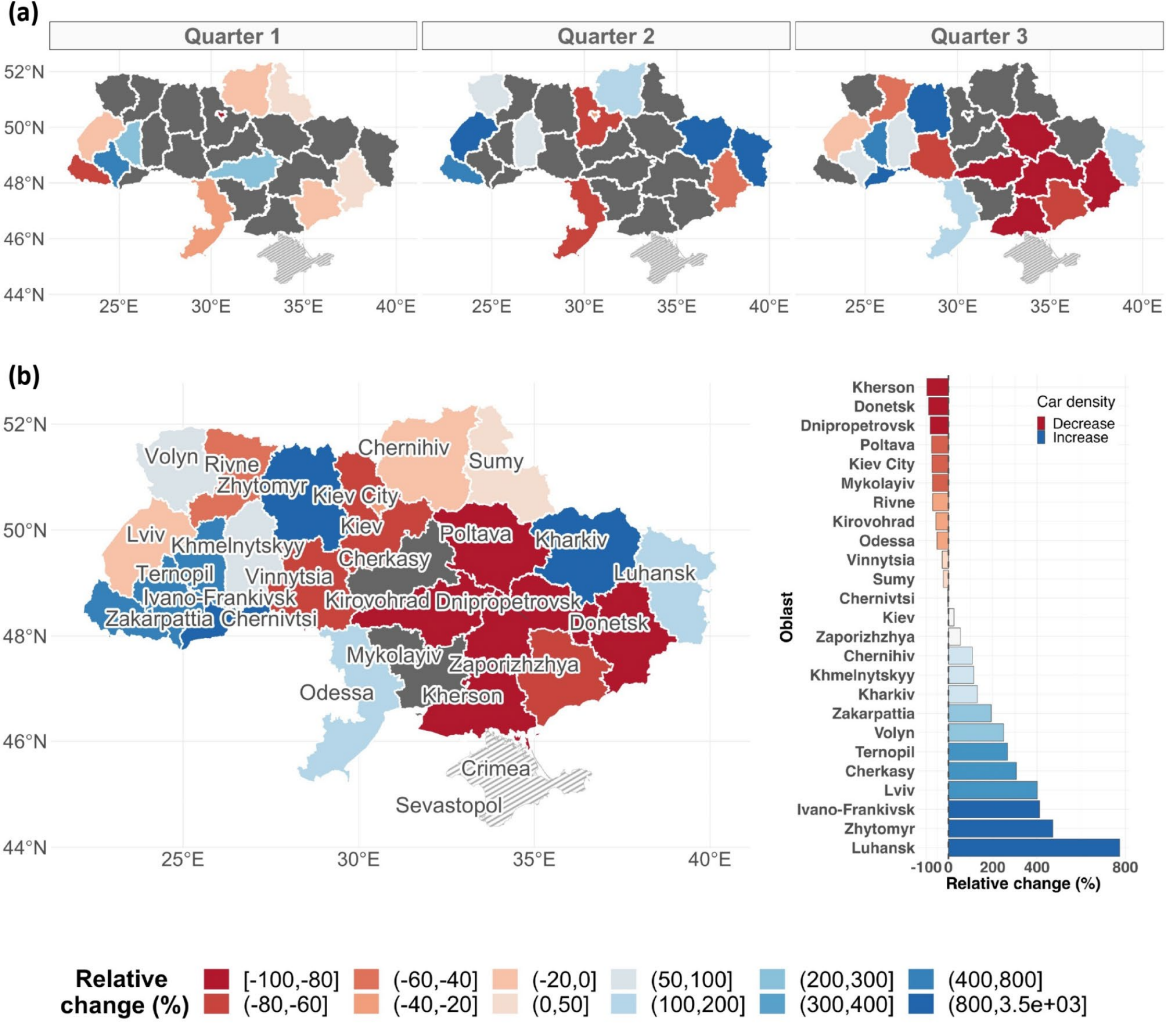
Change in average car density for all Ukrainian primary administrative units (Oblasts) during the first year of war (2022). Values reflect the percentage change in average car density after the start of the war (24 February) relative to the baseline (2019) for either quarterly (**a**) or yearly (**b**) temporal resolution. Oblasts colored in dark gray represent cases in which the relative change could not be calculated due to missing data for either or both years. The occupied territories of Crimea and Sevastopol were not considered in the current study (dashed areas). This figure was created using R software v.4.3.

Evaluating the car dynamics on a monthly resolution was largely infeasible, given the temporal data gaps within each city (see Supplementary Fig. S2). Nevertheless, trends in the car dynamics become more apparent at coarser temporal resolution and we therefore present the following results on a quarterly and yearly resolution to ease interpretation (Fig. 3).

At the quarter-of-year resolution, our results suggest an increasing and progressive trend of car displacement from East-to-West throughout the year (Fig. 3a). However, these results still suffer from considerable data shortages for some parts of Ukraine.

Analyzing the relative changes at the yearly level provided instead a more robust picture of the cars’ internal displacement (Fig. 3b). Except for Rivne, oblasts to the West were marked by a substantial increase in the number of cars. A particularly large increase was observed for Ivano-Frankivsk (412%) and Lviv (401%) (Fig. 3b). Oblasts in the central region of Ukraine depicted a more transitional state, as car density increased in some oblasts (e.g., Zhytomyr (472%), Cherkasy (307%), and Chernihiv (107%)), and decreased in others (e.g., Kherson (-98%), Poltava (-76%), and Kiev city (-75%))(Fig. 3b). The Eastern region, in contrast, was marked by a clear outflow of cars, with Donetsk (-90%), Dnipropetrovsk (-87%) and Mykolaiv (-72%) recording the largest drop (Fig. 3b). Kharkiv and Luhansk were an exception to this, given the number of cars increased for these two oblasts when contrasted to the number of cars circulating prior to the war (2019). Luhansk, indeed, recorded the highest increase among all Oblasts (+ 773%) (Fig. 3b).

At a high level, these aggregated results indicate a clear East-to-West movement, with some key oblasts suggesting potential cross-border movements (e.g., Odessa (Moldova), Ivano-Frankivsk (Romania), Lviv (Poland), Zhytomyr (Belarus), and Luhansk (Russia)) (Fig. 3b).

### Inferring IDPs from cars

Translating from shifts in car distribution to shifts in population distribution requires a model of the linkage between the two. To obtain such a model, we put fine-grained 2019 population counts in relationship to car counts from satellite imagery for the same year, and use the estimated relationship to predict population shifts along the first months following the start of the war.

**Fig. 4:**
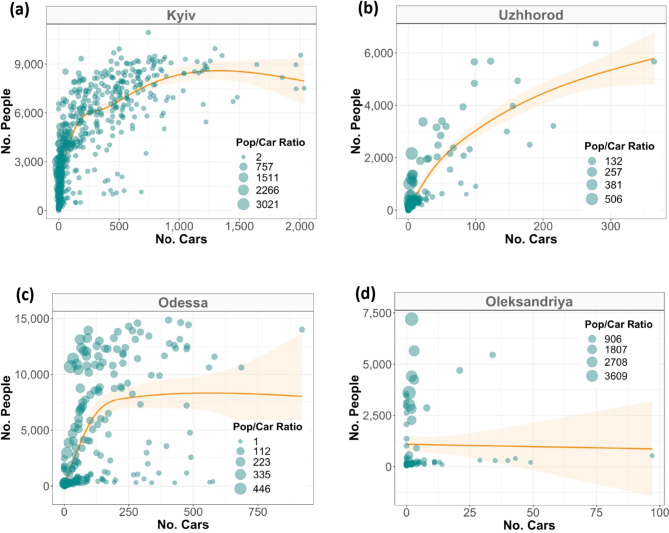
Relationship between the (gridded) average number of people and cars for four selected cities during the baseline year (2019). For most cities, the relationship is positive and non-linear akin to Kiev (**a**). Occasionally, the relationship is linear as for Uzhhorod (**b**), and rarely there is no (clear) relationship as in Odessa (**c**) or Oleksandriya (**d**). The orange smoothed function highlights the trend line from the GAM model bounded by its 95% confidence interval, with circle sizes scaled by the population/car ratio. Each data point assembles information from a unique grid cell (1 × 1 km) within the given city. For a complete overview, refer to Supplementary Figs. S3-S7.

There is a general positive link, i.e., areas with larger populations also have more cars visible in satellite imagery, which reinforces our initial hypothesis (Fig. 4). Oleksandriya was nevertheless an exception to this, as there is virtually no relationship between its population and the number of cars (Fig. 4). Curiously, there is variation in the exact curve linking population and car counts (Fig. 4 and Supplementary S3-S7). Furthermore, some highly populated areas were associated with a particularly low number of cars. Visual inspection suggests that such cases occurred mainly in dense residential areas where cars could be parked in closed spaces such as garages. It is likely that other factors such as differential rates of car ownership, street parking availability, and access to and quality of public transportation could also explain this variation.

To model this car-to-people relationship, we explored two approaches with distinct levels of complexity. In the first approach, we assumed that the 2019 car-to-people ratio still applies in the following years while remaining constant within areas; hereafter the *ratio method*. That is, if for a given area in a city the number of visible cars drops by, say, 40% compared to 2019, we assume that the population in that area has also dropped by 40%. This approach is intuitive, but it has a risk of “overfitting”, and it always assigns a population count of zero to areas without visible cars. Moreover, an additional limitation is that the fixed car-to-population relationship fails to account for changes in urbanization levels, an aspect that is likely to occur in the long-run for any city or areas within the city. We nevertheless deem that this effect was negligent in our case study, given the narrow time window (2019–2022) which also involved two years of heavy economic recession that was driven by the COVID-19 pandemic.

In the second approach, we relied on Generalized Additive Model (GAM) as it provides a more flexible function to estimate the cars-to-people relationship; hereafter the *regression method*. Unlike the ratio method, this approach tends to overestimate the population size, as it typically assigns the function’s average to all areas, including uninhabited areas. By using these two distinct methods, we can ultimately derive lower and upper bound estimates, and thereby provide a proxy for uncertainty in our population size estimates.

Overall, we could estimate IDPs for 43 out of the 61 evaluated cities. The remaining cities did not have any comparable month between the baseline (2019) and the other two years (2020, 2022), and were thus not considered for further analysis. For an in-depth overview on our above data analytical assumption, we refer the reader to the Material & Methods section. For the majority of applicable cases, our findings indicated that the population was larger during the first COVID-19 year than during the conflict year (e.g., Bila-Tserkva, Donetsk and Odessa; see Supplementary Figs. S8 and S11), as one might expect.

**Fig. 5:**
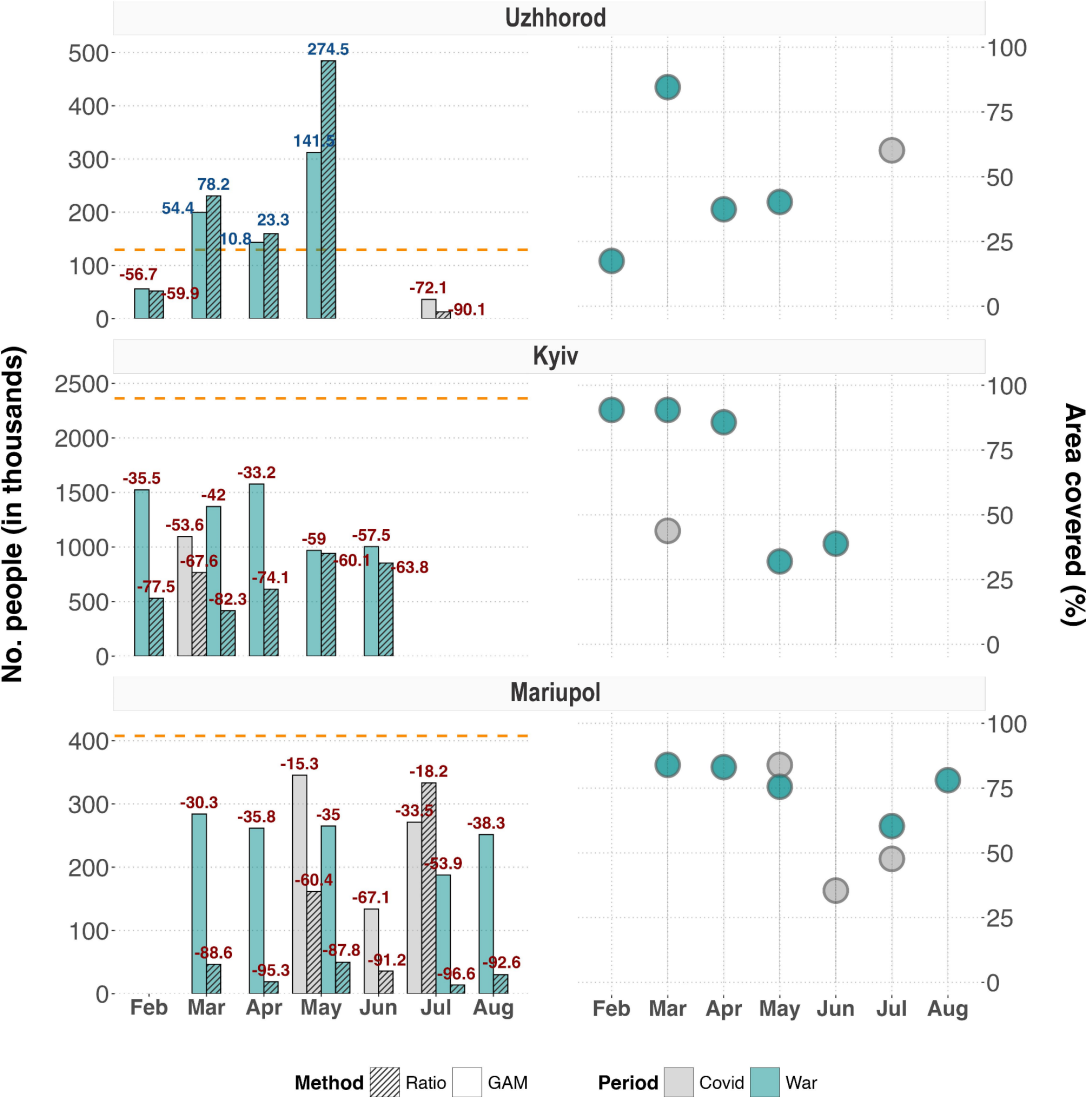
Predictions of internally displaced people across three different cities (left panels). The orange line marks the pre-war population size (2019), from which relative change has been derived for the applicable months in either 2020 (first COVID-19 year, gray bars) or 2022 (war year, turquoise bars). Numbers on top of each bar denote the relative population change (in %), with colors reflecting either an increase (blue) or decrease (red). Dashed and plain bars distinguish the two tested prediction methods: linear ratio (dashed) and Generalized Additive Model (GAM, plain). Right panels depict the percentage of area covered by the satellite images underlying a given month relative to the city’s area of interest (AOI). Note that the larger population drops/increases for some cities and months should be interpreted with additional care, as it could be an artifact induced by the smaller spatial extent that is reflected by the underlying satellite images. The models for Uzhhorod, for example, predicted a population increase of up to 23% in April 2022 (left panels). This number is nevertheless likely underestimated, as the collection of satellite imagery for the given month covered less than 50% of the city’s extent (right panels). For the full set of results, refer to Supplementary Figs. S8-S12.

Although the population estimates could differ substantially between the two prediction methods (i.e., ratio and regression methods), their general trend remained consistent (Fig. 5 and Supplementary S8-S12). While the ratio method predicted usually much larger population drops (or increases), the regression method resulted in milder and less extreme predictions. Following the ratio method, for example, more than 88% of Mariupol’s population emigrated in March 2022. These fluctuations are dampened when considering the estimates from the regression method, which predicted a decrease of only 30%. Such discrepancies are naturally expected, given that the GAM approach adds the average population of the baseline year into its calculation, unlike the ratio approach which considers only the fixed population-to-car ratio.

For a handful of cities, some months in the regression approach were marked by either suspicious population sizes (e.g., Berehove and Mamalyha in Supplementary Figs. S8 and S10, respectively), or differing population trends between the two prediction methods (e.g., Donetsk, Luhansk and Shehyni in Supplementary Figs. S8, S10 and S12, respectively). This likely results from poor model fit, violation of one or more model assumptions, or because the number of cars during the conflict year was simply far outside the model’s prediction range.

The results from the GAM approach generally revealed a good performance in terms of goodness-of-fit, as most of the evaluated cities (25/43) displayed a coefficient of determination above 60% (see Supplementary Table S1), and followed reasonably well the evaluated model assumptions (refer to Supplementary S13 for an example). This means that the city-specific baseline population sizes could be generally well estimated by the number of cars, and the model is as such suitable for predicting population during the war period. For models outside these conditions, we caution against any solid conclusion.

Irrespective of the prediction method, our results revealed similar trends in terms of people displacement across cities and oblasts. In line with the raw car dynamics reported previously, the current results also suggest an East-to-West movement of population (Fig. 5 and Supplementary Figs. S8 - S12). In the first months following the start of the war (March-April), cities in the West, such as Uzhhorod and Ivano-Frankivsk, also showed a substantial increase in the number of people compared to their pre-war population (Fig. 5 and Supplementary Fig. S9). The population of Ivano-Frankivsk increased substantially compared to all other western cities, with estimates ranging between +157% - +623% above the pre-war population size (see Supplementary Fig. S9). In contrast, cities in the East and more central regions, such as Kiev and Mariupol, mostly saw an outflow of people (Fig. 5 and Supplementary Figs. S8 - S12). Among those, the cities of Zaporizhzhia and Kherson were marked by the largest population drop, where the ratio method predicted a population decrease of more than 90% for both cities (see Supplementary Figs. S10 and S12).

**Fig. 6:**
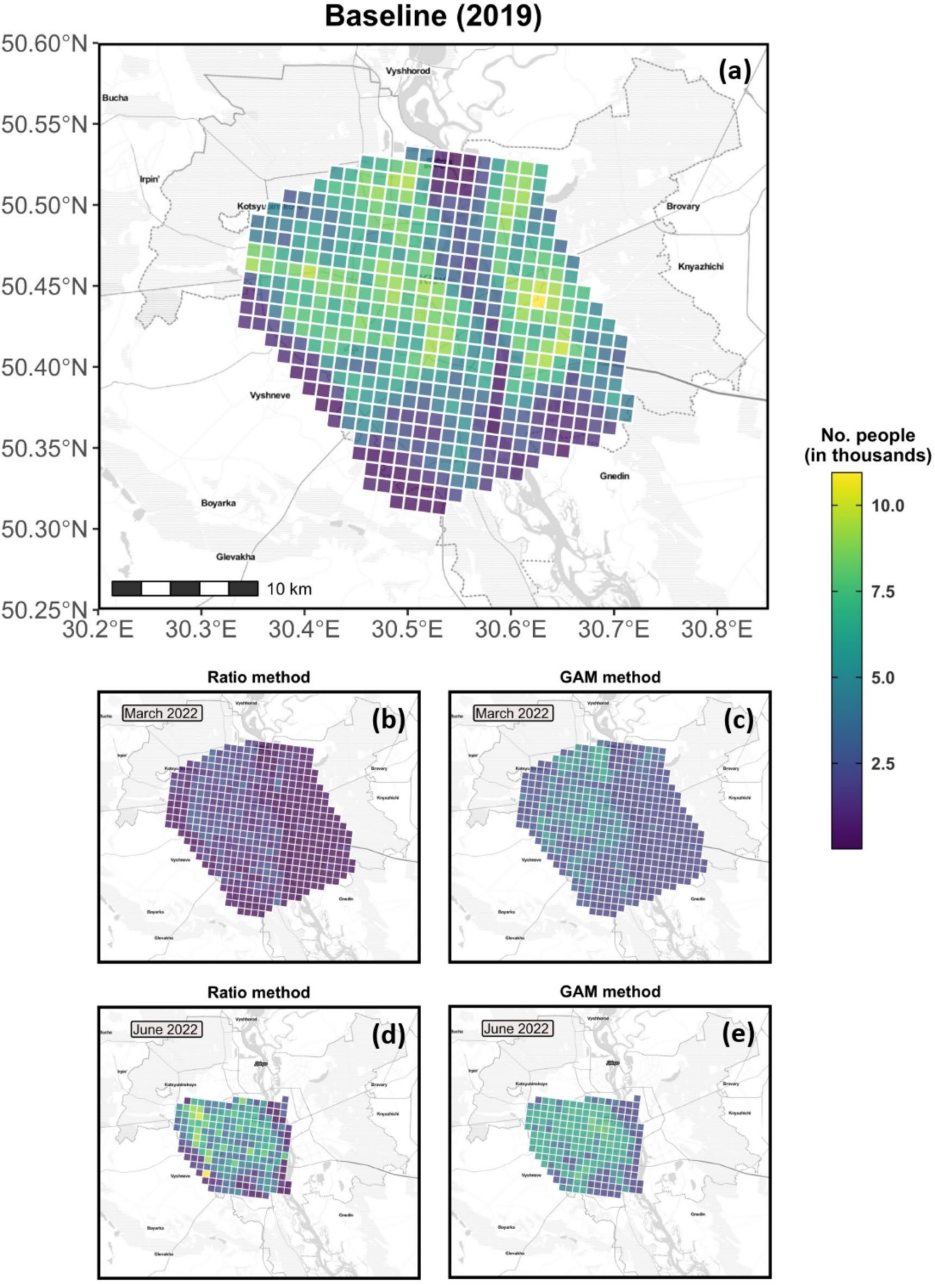
Gridded population for Kiev city, with each grid cell measuring 1 × 1 km. The baseline population (**a**) was retrieved from WorldPop’s database, whereas the population for March and June 2022 were predicted through either the Ratio (**b**,**d**) or the GAM method (**c**,**e**). Note that the satellite images underlying the month of June 2022 covered only a fraction of the city’s AOI (**a**), which is also denoted in the right panels in Fig. 5. To interpret the present figure in relative terms, refer to Supplementary Fig. S14.

Apart from the city-level comparisons, it might be of general interest to understand the displacement dynamics at the sub-city scale. Figure 6 shows an example of the predicted population for Kiev city for two different months in 2022, with Supplementary Figure S14 providing additional support by showing the results in relative terms. Regardless of the prediction method, our results clearly show an extensive emigration of its residents in the first month following the war (March), with the eastern side of the city marked by a larger population drop than the western side (see mid panels in Fig. 6). This picture changes completely three months later (June), when the eastern portion of the city seems to have recovered most of its initial population (see lower panels in Fig. 6).

## Discussion

Estimating population displacement at the subnational level is inherently challenging due to its dynamic nature and the irregular collection of primary data. Our analysis demonstrates the potential that very-high-resolution satellite imagery holds for monitoring car-based internal displacement. The importance of such methods is likely to increase, as climate change and political and economic instabilities are bound to lead to additional displacement pressure worldwide in the years to come^3,29,30^.

We investigated two different approaches to predict IDPs and despite the occasional discrepancies, some clear movement patterns could be traced at both subnational and sub-city level. These approaches could be improved further if situation-specific information on the mode of (escape) transport is available, e.g. from post-displacement surveys. However, groups such as the “caminantes”, who are leaving Venezuela on foot, will be invisible to our method. Similar challenges also apply to the use of mobile phone data, as the exact phone-to-person link is context-dependent and parts of the population might have several mobile devices per person, while others have none. Connectivity can be additionally affected by power shortages or destroyed cell towers, and thus blurring the IDP dynamics.

Following our findings, most of the IDPs originated from the eastern region due to the decreased population estimates. Oblasts to the west, in contrast, were frequently associated with increased population estimates, suggesting as such their role as host regions. At the high-level comparison, these findings corroborate with the trends reported by the International Organization for Migration (IOM)^6^, and are further supported by those from Rowe et al.^8^ and Leasure et al.^10^. Comparing these estimates in absolute terms is nevertheless intractable, given the differences in spatial and temporal granularity, including the data source underlying the reference year. In the absence of ground-truth data with which our population estimates could be possibly validated, such high-level data triangulation remains the only option at the time being.

While our study focuses on one country, Ukraine, and one satellite image provider, Maxar, we believe that the computer vision approach for detecting and counting cars will generalize to other geographic contexts that are similar in terms of car, building, road types, as well as to other providers of very high-resolution satellite imagery (30–50 cm). The benefits of such an approach are multifold for both the humanitarian and government sector, going from reduced life-treating risks for the on-ground enumerators to the rapidity at which population estimates can be generated as a response to the frequent imagery update. Besides, a major advantage of the current method is its transparency, unlike the black-box, and often biased, algorithms employed by social media platforms such as Meta.

Nevertheless, our framework holds some fundamental limitations that could hamper its broader diffusion. To start with, very-high-resolution imagery such as the ones used herein are not cheap to procure and require staff with technical skills to acquire, process, and analyze them^12^. This might impose a barrier for low- and middle-income countries, who already suffer from long-lasting funding restrictions. Additionally, it can take time to establish partnerships between governments and imagery providers.

Current computer vision techniques also come with limitations that can jeopardize the accuracy and generalization of any similar framework^31,32^. A typical constraint of AI-based models is the lack of ground-truth data to perform their formal validation in the real world^33,34^. In the absence of a dedicated database, it is necessary to employ expert human annotators to manually label the images. This is not only costly and time-consuming, but often infeasible in the context of humanitarian crises which require timely responses.

Some other constraints are intrinsic to the satellite images themselves. In our brief experiment, we showed the effect of weather- and cloud-based obstructions on car detectability, including imagery-related features such as color, off-Nadir angle, and image resolution (see section ”Understanding the effect of imagery features on car detection”). Given its unknown impact on population estimators, we largely encourage future research to investigate further the impact of such aspects on object detectability, and most importantly the use of high-resolution synthetic aperture radar (SAR) imagery as a potential remedial solution to the limiting RGB-based images (https://umbra.space/).

Data sparsity was also a key limitation in the present study, mostly as a result from the pre-collected archival Maxar imagery. Despite the better spatial coverage and cadence of the freely available Sentinel-2 images, its spatial resolution of 10 m per pixel prevents the detection of small objects like cars. Most cars typically occupy less than a pixel, making it hard to distinguish them from the background. For this reason, we resorted to Maxar images which offer higher spatial resolution (i.e., 0.3–0.5 m per pixel), yet come at the cost of lower spatial and temporal coverage as our analysis relied on pre-collected archival imagery, rather than on-demand task-based imagery.

However, it is worth to highlight that the number of earth observation satellites has improved rapidly, jumping from 789 in Aug 2020 to 1238 in May 2023^35^, with a further increase expected. Furthermore, the cost of tasked-based imagery has dropped to under USD 1000 per request^36,37^, down from over USD 10,000 a few years back, with further price drops expected. With improved data availability, it is likely to open some niche for exploring more advanced and robust statistical models to predict population shifts from observed car displacements, including, but not limited to, spatial and state-space models^38,39^.

Beyond the aforementioned cases, there remains the fundamental research challenge of tying the number of displaced cars to the number of displaced people. Behavioral changes induced by the war could likely affect the pre-war link between the number of visible cars and the number of people living in a given area. For example, one might assume that, when fleeing, the cars could be more packed. The drop in cars could be thus an underestimation of the drop in population. Conversely, the cars that remain might well become more hidden/protected, overestimating as such the population displacement.

While our research focuses on computational methods for estimating internal displacement, it is important to acknowledge that such work does not happen in a political vacuum. In particular, there is a risk that any migration-related technology will be used to curb and restrict migration, rather than benefiting migrants^40^. And even if used with good intentions in a humanitarian context, more data could add noise and distract, while also creating privacy issues^41^. Despite these valid concerns, we believe that if developed and deployed responsibly through academic-humanitarian partnerships, satellite-based estimates can benefit displaced populations, reducing privacy risks related to the use of individual data, such as mobile phone traces.

## Material & methods

### Car detection and counting from satellite images

Automatic detection of small objects such as cars from satellite images is a difficult task, especially at a global scale, due to the diverse nature of environments across geographies, climate zones, and seasons. To tackle this challenge, many methods have been proposed using traditional approaches such as training classifiers (e.g., Support Vector Machines (SVMs)^42^ and Random Forests (RFs)^16^) over handcrafted features (e.g., Local Binary Patterns (LBP)^43^, Histogram of an Oriented Gradients (HOG)^44^, and Scale-Invariant Feature Transform (SIFT)^45^)^46–48^. However, more recent approaches leverage on large labeled image collections such as xView^49^, DOTA^50^, DIOR^51^, and FAIR1M^52^ by using various deep learning architectures based on Convolutional Neural Networks (CNNs)^53–56^. The CNN-based approaches have achieved better performance thanks to the capacity of deep neural network architectures to extract and learn object characteristics within an end-to-end framework^51,57–64^.

In this study, we employed the state-of-the-art ensemble CNN framework proposed by Minetto et al.^65^, which ranked third place in the xView challenge (http://xviewdataset.org/), the most advanced benchmark for object detection in satellite images, organized by the US Defense Innovation Unit Experimental (DIUx) and the National Geospatial-Intelligence Agency (NGA). The ensemble model is designed by combining two baseline Single Shot Multibox Detectors (SSD)^55^ with various data augmentation strategies adopting different scales, overlaps, and thresholds in order to ensure better scale invariance and detection accuracy for small vehicles. More technical details about the method can be found in Minetto et al.^65^.

Different than the reference work^65^, we focused particularly on the *small car* class and filtered the final car detections using a higher confidence threshold of 0.45 which we tuned by a sensitivity analysis experiment as follows: We took a sample of 3000 images with a total of 19,000 ground-truth car annotations and evaluated the car detection model’s output performance by computing *F*_*β*_ score as in Eq. (1) with *β* = 0.5 while increasing the confidence threshold value from 0 to 1 with a step size of 0.05.

1 $$\:\begin{array}{c}{F}_{\beta\:}=\left(1+\:{\beta\:}^{2}\right)\frac{Precision\:\times\:Recall}{\left({\beta\:}^{2}\times\:Precision\right) + Recall}\:\end{array}\:$$

In this analysis, we chose *β* = 0.5 to put more weight on precision than recall of the model, and hence, focused on minimizing false-positive car detections which is critical for our use case. Supplementary Table S2 summarizes the performance achieved by the model in terms of Precision, Recall, and *F*_0.5_-score across varying confidence thresholds. The maximum *F*_0.5_-score was achieved as 0.4776 when confidence threshold was 0.45 as highlighted in Supplementary Fig. S15. At this threshold value, the precision and recall scores of the model were recorded as 0.5578 and 0.3032, respectively, according to Supplementary Table S2.

### Study region and areas of interest

Our study region comprised all primary administrative units, i.e., oblasts, in Ukraine, except for the occupied territories of Crimea and Sevastopol. Within each oblast, we selected the two most populated urban areas, in addition to other strategic areas located at the border checkpoints which were used as escape routes; hence, where humanitarian efforts could be optimized.

The selection of urban areas was based on the open-source gridded population data retrieved from WorldPop, which is regularly updated and curated by the WorldPop Research Group at the University of Southampton^66^ (https://www.worldpop.org). For the purpose of this study, we downloaded the geospatial layer tailored specifically for Ukraine^67^. This data provides information for the latest available year (2020) at a resolution of 100 m, and was produced through the top-down constrained method (for more details, see Stevens et al.^68^ and WorldPop^69^). We then aggregated the population data at the secondary administrative units, i.e., Raions (districts), and ranked them within each oblast. Ultimately, we selected the cities belonging to the top-two most-populated Raions in each oblast and manually defined a boundary area around the identified cities.

To define the strategic areas, we relied on information provided by the Humanitarian Data Exchange (HDX) database (https://data.humdata.org/). HDX is an open-source platform managed by the United Nations Office for the Coordination of Humanitarian Affairs (OCHA), providing over 20,000 datasets spanning 250 locations. We specifically retrieved information regarding international border crossings of Ukraine, for which the most up-to-date data was downloaded at the development of this study (June 2022). This dataset nevertheless does not specify the type of border crossing and can include categories such as railway and water crossing. In order to keep locations that can be only crossed by vehicles, we further intersected this data with information provided by the Ukrainian State Border Guard Service (https://dpsu.gov.ua/).

This process resulted in 61 areas of interest (AOI) with varying sizes, whereby each oblast included at least two AOIs (Fig. 1). All-together, these 61 AOIs covered a total area of 13.496 km^2^(see Supplementary Table S3). For simplicity, we will henceforth refer to each AOI as ‘city’.

### Satellite imagery collection and processing

Reliable detection of small objects such as cars requires access to very-high resolution satellite images. Although there are a number of free satellite imagery providers (e.g., Sentinel data), they all come at the cost of coarser spatial resolution (> 10 m). For this reason, we used pen-sharpened natural color images from the Worldview and GeoEye series of satellites operated by Maxar Corp, which currently provides images at a spatial resolution of up to 30 cm.

Through the SecureWatch Platform (https://securewatch.maxar.com/myDigitalGlobe), we searched the archive for images taken between January 2019 and September 2022. We included images from the years before the war so that we could contrast more reliably the car dynamics before and during the war. Moreover, we started from January 2019 because car dynamics might have likely changed during the COVID-19 outbreak and would, as such, not provide a reliable baseline for comparison. The list of available satellite images was further filtered to retain images with (i) RGB color, (ii) less than 20% cloud coverage, and (iii) a Ground Sampling Distance (GSD) of at most 0.5 m (i.e., images at 0.3 m, 0.4 m, and 0.5 m resolution). Note that GSD refers to the distance between two consecutive pixels in an image measured on the ground. The smaller the value of GSD, the higher the spatial resolution of the image and the more visible the details of small objects such as cars.

After this initial search, we proceeded to download satellite imagery for each AOI, for every day (where available) in the above-mentioned period. For each AOI, we downloaded an image for a given day only when there were one or more images available covering at least 1% of the AOI’s area on that day. If there was only a single satellite image available for that day, we downloaded the portion of the image that covered the AOI. If there were more than one satellite images available on that day we downloaded a single stitched image as follows. The available satellite images were sorted based on area of the AOI they covered and the image covering the most area was downloaded first; this procedure was then repeated with the remaining images for the still-uncovered portions of the AOI. These different image portions were then stitched together into one final image. We downloaded a total of 1009 daily image snapshots across all 61 AOIs. Most of these snapshots (77.7%) were composed of a single satellite image with the remainder being stitched from two or more images as described above. The bulk of the images were taken between 8 AM and 9 AM UTC (see Supplementary Fig. S16).

### Data post-processing

Preliminary assessments revealed that certain images were associated to an outstanding number of car detections, while others only to a few or even none. Further investigation indicated that the outstanding detections were mostly related to false-positives. Conversely, images with zero or few detections were associated to cases in which the imagery was either fully obstructed by dense cloud and/or haze layers, or where it covered only a very small fraction of the AOI. To avoid confounding noise, hence misleading the results, we carried out three additional data filtering processes described as follows.

#### False-positive filtering

As any ML model, the car detection and classification algorithm used herein is not immune to false-positives. Prior investigation revealed that the CNN model detected cars at places in which their occurrences are unlikely or even impossible (e.g., water bodies, crop fields and forests; see Supplementary Fig. S17). To attenuate these misdetections, we juxtaposed the detected cars with some of the spatial attributes retrieved from the OpenStreetMap (OSM) database and filtered out any detection located within the unlikely areas.

OSM is a crowd-sourced database that provides physical features from all over the world^70^. The data is organized by tags, that correspond to a key-value pair describing the feature’s characteristics. Based on the official map attributes exposed in the Wiki OSM webpage (https://wiki.openstreetmap.org/wiki/Map features), we selected five primary keys to compose our standard tag: landuse, place, natural, leisure, and aeroway. Each key was then paired to a list of values, defining ultimately areas such as rivers, forests, farmland, parks, and railways. For a detailed list of the selected tags, we refer to Supplementary Table S4.

To retrieve the geospatial layers related to our list of selected tags, the osmdata R-package was used^71^. The package essentially downloads OSM data through overpass API queries, where each query runs within a bounding box area. In our case, we ran the query for all 61 AOIs (i.e., bounding boxes), and stored the AOI-specific data in the format of shapefiles. Any cars detected on those layers were considered as false-positives, and hence, removed from our database.

#### Population filtering

Most of the downloaded imagery covered only a fraction of the AOIs. In some cases, the covered fraction was very small and typically did not overlap with the core extent of the urban area (see Supplementary Fig. S18). We deemed such images as unrepresentative of the city’s core dynamics, and consequently removed them from the analysis. Specifically, we classified as unrepresentative any imagery covering less than 50% of the population distribution. For this purpose, each imagery was assigned to a distribution index, calculated as follows:

2 $$\:\begin{array}{c}{p}_{i,\:s}=\left(\frac{\sum\:{N}_{i,\:s}}{\sum\:{N}_{s}}\right)\:\times\:100\:\end{array}\:$$

where *p*_*i, s*_ represents the population fraction covered by the imagery *i* relative to its AOI *s*, and *N*_*i, s*_ and *N*_*s*_ indicate the number of people within the imagery and AOI, respectively. The index ranges between 0 and 1, with larger values corresponding to stronger representativeness of the full population distribution. Note that to compute the indices, we used the same gridded population data as presented in section “Satellite imagery collection and processing”.

#### Cloud and haze filtering

Some images were associated to very low car detections (N < 10), or even none in the more extreme cases. In order to make reliable inferences on the car dynamics, it is imperative to distinguish false from true zeros; or, alternatively, low occurrences. False zeros/low occurrences are of particular concern, as they may arise from images obstructed by factors such as clouds, haze and pollution.

Despite the initial imagery request was limited to cases in which the cloud coverage did not exceed 20%, it does not warrant that the AOI will be cloud free. This is because MAXAR calculates the cloud coverage on the entire image tile, instead on the AOI (i.e., subset of the provided tile). Most of the zero/low occurrences detected in our data were due to images that were fully or partially covered by a dense cloud/haze layer. We therefore removed all images that were fully obstructed, while partially obstructed images were removed only if the cloud(s) blocked the bulk of the city. Note that for the latter aspect, images were visually inspected as there is no gold solution to filter cloud-obstructed images.

### Data analysis

#### Evaluating the spatial and temporal dynamics

To investigate the potential internal migration dynamics before and during war period, we evaluated first the temporal car dynamics for each city whenever applicable. Data scarcity prevented the evaluation on a daily basis, and thus monthly average of car density were computed for all cities. Moreover, to examine regions that experienced an increase/decrease in the number of cars during the conflict year (2022), we calculated the change in average car density relative to the baseline year (i.e., 2019). To draw a country level picture, relative changes were calculated at the level of primary administrative units (Oblasts). For Oblasts with two or more cities, this means that the average car densities were further averaged across the cities.

#### Inferring IDPs from cars

We assumed that spatial and temporal changes in the number of cars could reflect potential migration of Ukrainian citizens across the country. Thus, we specifically propose to estimate war-induced IDPs from historical population-to-car trends incurred during the baseline year (i.e., 2019). In the absence of ground truth data, we used WorldPop’s gridded population data for the year 2019 to reflect more realistically the baseline population.

Overall, two distinct methods were used to estimate the relationship between pre-war population and cars. Specifically, we used the (i) linear ratio and (ii) regression method^72^, which differ in terms of complexity and assumptions. Whereas the ratio method assumes that the population-to-car relationship remains invariant through time within the given geographical area, the regression method relaxes this assumption by including various levels of complexity, such as non-linear and/or spatially- and temporally-dependent relationships. By using two distinct methods, we can provide lower and upper bound estimates, and hence, a proxy for uncertainty.

The forecasting should be ideally conducted on a daily basis, since such fine-grained estimates would be more relevant during the acute phase of the humanitarian crisis. However, such high temporal resolution does virtually not exist in the context of historical satellite imagery, and therefore, IDPs were predicted on a monthly basis.

We initially constructed a 1 × 1 km spatial grid for all cities through the sf R- package^73^. The total number of cars and people were then computed at the grid cell level for each city-specific satellite imagery taken in the years 2019 (baseline year), 2020 (first COVID-19 year), and 2022 (first conflict year). We used the results from the first COVID-19 year to contrast with those from the conflict year and assure that the latter results were realistic given past trends. That is, we should expect that the population during the COVID pandemic is larger than the population during the war period.

To avoid any hidden and potentially misleading seasonality effect that might be induced by the extensive temporal gap, we identified and selected only cities presenting one or more matching months between 2019 and at least one of the two other years (i.e., 2020, 2022). Monthly averages of the number of cars and people were then computed at the grid cell level for each city and year. Next, for the reference year (i.e., 2019), we pooled information from all months and calculated the average number of cars and people for each city and grid cell therein. These averaged values were subsequently used to estimate the pre-war relationship between population and cars for each of the prediction methods (i.e., linear ratio and regression model). Because the extension of the spatial grid might differ between the considered periods (e.g., Fig. 6), we assured that all predictions were conducted on the matching grid cells only.

In the ratio method, we specifically calculated the proportion of the number of people *y* relative to the number of cars *x* as follows:

3 $$\:\begin{array}{c}{r}_{s,\:z}=\left(\frac{{y}_{s,\:z}}{{x}_{s,\:z}}\right)\:\end{array}\:$$

where *r*_*s, z*_ is the population-to-car ratio for city *s* and grid cell *z* for the reference year. Certain grid cells may register zero cars, whereby the ratio cannot be computed. For such cases, we calculated the global median of the ratio and borrowed the computed value for the affected cells. The grid-level ratios from the reference year were then used as multiplying factor to predict the city-specific population from the average number of cars that were calculated for the matching months in 2020 (COVID-19) and 2022 (war).

In the regression method, in contrast, we estimated the baseline population car relationship through a Generalized Additive Model (GAM), as prior examination indicated an asymptotic trend when plotting the two variables irrespective of the grid cell index. Unlike the ratio method, GAMs are a class of statistical models in which the uncertainty can be retrieved in both estimation and prediction phase. These models constitute a powerful extension of GLMs (Generalized Linear Models), whereby the linearity assumption of the predictor(s) can be relaxed through smoothing functions^74^.

Similar to GLMs, the response variable *Y* (herein no. of people) follows a probability distribution from the exponential family (herein Poisson), with the mean *µ* = *E*(*Y* ) linked to an additive non-parametric predictor η (herein no. of cars) through a link function *g*(.), such that *g*(*µ*) = *E*(*η*). For a given city, and month-year, the model can be thus simplified as:

4 $$\:\begin{array}{c}{Y}_{i}=exp\left({\beta\:}_{0}+f\left({X}_{i}\right)+\:{\epsilon\:}_{i}\right)\:\end{array}$$

where $\:{\beta\:}_{0}$ is the intercept (i.e., global average of cars), $\:f$(.) the cubic spline function, ε the error term, and *i* the grid cell index.

All GAM models were fitted through the mgcv R-package^75^, and model assumptions checked visually through the mgcViz package^76^. The parameters from the fitted models were ultimately used to predict the grid-level population for the matching months in 2020 and 2022 based on their average number of cars, akin to the ratio method.

For both approaches we calculated the change in population size relative to the baseline population. Computations were performed on both grid-cell and global level, where the latter was calculated by summing up the grid-level populations.

#### Understanding the effect of imagery features on car detection

It is paramount to understand the influence of imagery-related characteristics on the CNN’s model capacity to detect cars, as it influences the IDP estimates. Cloud obstruction, sun elevation and off-Nadir angles, for example, can influence the geometry of the image and hence accuracy of the object detection^77,78^. Thus, to examine the overall effect of these features on car detection, we conducted a Generalized Linear Model (GLM) through the stats R-package. The number of cars were standardized by the area of the given imagery (i.e., car density), log-transformed, and modelled via a Gaussian probability distribution. Among the imagery features, we evaluated the effect of image resolution (categorical), off-Nadir angle (numeric), sun elevation (numeric), cloud coverage (numeric), and presence of snow (categorical). Model assumptions were visually assessed to evaluate the residuals’ normality, homoscedasticity and independence.

**Fig. 7:**
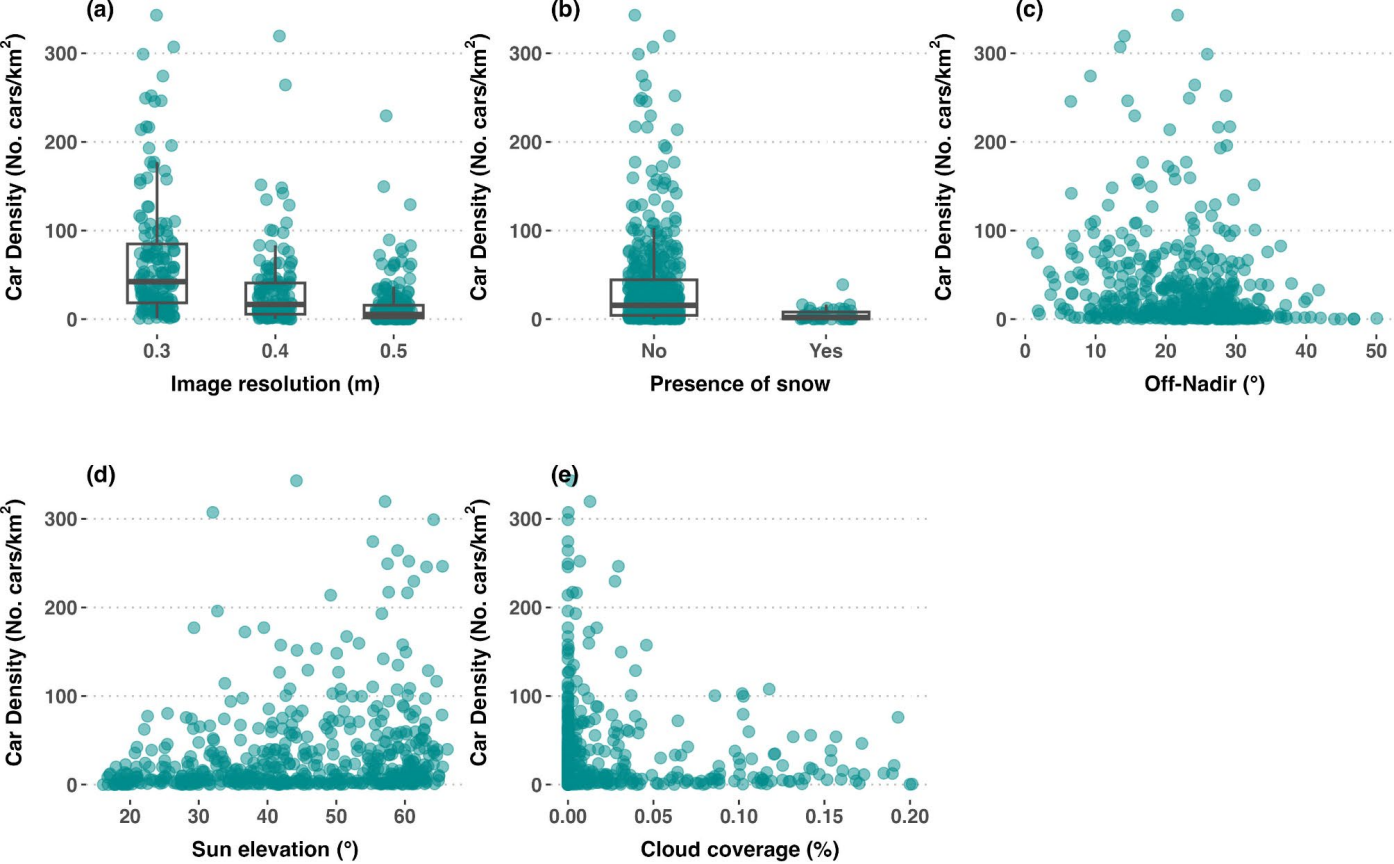
Car density expressed as a function of (**a**) image resolution, (**b**) presence of snow, (**c**) off-Nadir angle, (**d**) sun elevation angle, and (**e**) percentage of cloud coverage.

Our results revealed that all tested covariates significantly affected the car detection, except the cloud coverage (Table 1). Higher image resolution was generally associated with higher densities of detected cars (Table 1; Fig. 7a and Supplementary Fig. S19). The presence of snow was negatively related to car densities, meaning that a higher number of detected cars tended to be associated with non-snowy days (Table 1; Fig. 7b). Supplementary Figure S20 shows an example of the impact of snow on car detection, reducing the contrast between cars and their surroundings, making it more difficult for the model to discern cars in the image.

**Table 1:** Statistical summary of the generalized Linear Model (GLM) conducted to test for the effect of imagery-related features on car density.

Parameters	Estimate	2.5%	97.5%	t-value	*P*-value
Intercept	3.44	2.85	4.02	11.55	< 0.05
Image resolution − 0.4	− 1.05	− 1.37	− 0.73	− 6.45	< 0.05
Image resolution − 0.5	− 1.90	− 2.20	− 1.61	− 12.59	< 0.05
Snow presence - Yes	− 1.67	− 2.16	− 1.18	− 6.72	< 0.05
Off-Nadir	− 0.03	− 0.05	− 0.02	− 3.99	< 0.05
Sun elevation	0.02	0.01	0.03	4.49	< 0.05
Cloud coverage	− 1.74	− 4.55	1.07	− 1.22	0.225

Estimated parameters are on a log-scale and include the 95% confidence interval, *t*-values, and *P*-values expressed at 5% of level of significance.

The off-nadir and sun elevation angles had an antagonic effect on car detection, both of which were directly related to the occlusion of important infrastructure such as roads and parking lots by buildings or their shadows, and thus, affecting car detectability (Supplementary Figs. S21 and S22). Whereas car densities were larger at smaller off-nadir angles and with maximum detectability around 25° (Table 1; Fig. 7c), car densities were higher towards larger sun elevation angles (Table 1; Fig. 7d).

Surprisingly, no significant impact from cloud obstruction could be detected from the present data. Although lower car densities seemed to be associated to images that were more heavily obstructed by clouds (Fig. 7e), the overall effect was not statistically significant (Table 1). It is noteworthy that all model assumptions were reasonably met, i.e., residual normality, homoscedasticity and independence. We refer to Supplementary Figure S23 for a visual overview of the model’s diagnostics.

## Electronic supplementary material

Below is the link to the electronic supplementary material.

Supplementary Material 1

## Data Availability

All data and codes underlying the findings of the present study are available on the first author’s GitHub repository (https://github.com/mcruf/IDP_UKR). We nevertheless note that the original Maxar satellite images cannot be openly shared due to confidentiality reasons. These can, however, be made available from the first author upon prior permission from Maxar.
