# Supplementary material for: Estimation of internal displacement in Ukraine from satellite-based car detections
Source: Sci Rep. 2024 Dec 30;14:31638. doi: 10.1038/s41598-024-80035-8 (PMC11685971; doi:10.1038/s41598-024-80035-8)
Supplement: Supplementary file 1 — Supplementary Material 1 [file 41598_2024_80035_MOESM1_ESM.pdf]

# - Supplementary Information -

Estimation of Internal Displacement in Ukraine from Satellite-Based Car Detections

Marie-Christine Rufener, Ferda Ofli, Masoomali Fatehkia, Ingmar Weber

Supplementary Figures

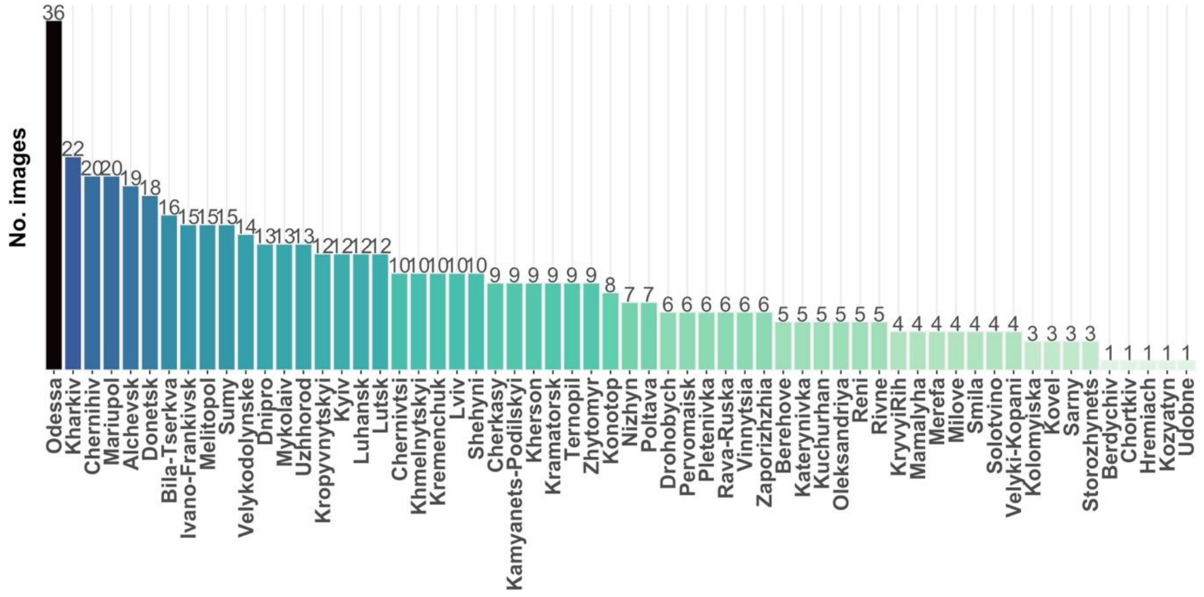

Figure S1: Number of satellite images per city after the data post-processing pipeline (n = 534 images).

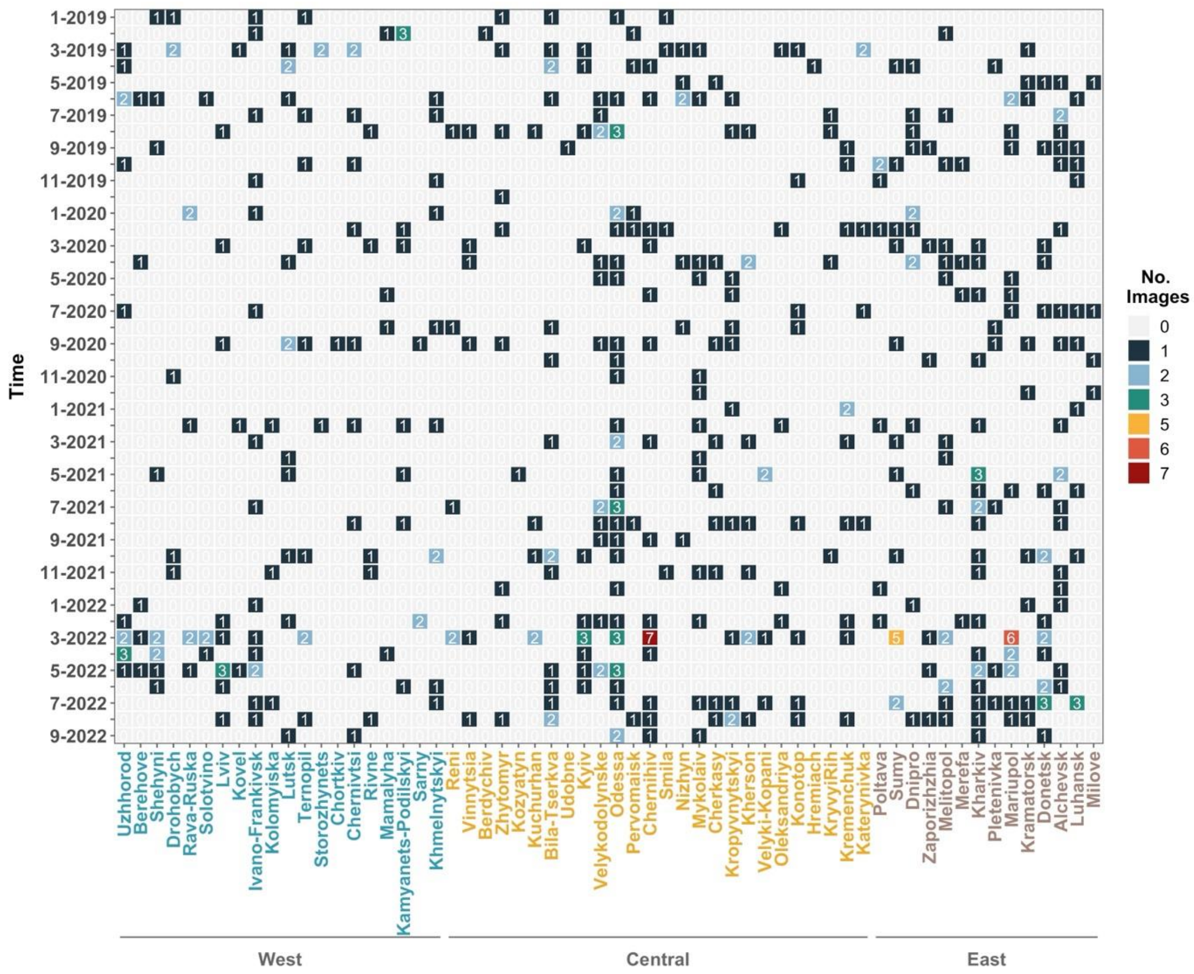

Figure S2: Data availability across space and time after the data post-processing pipeline (n = 534 images), with cities arranged from West to East. The color scale reflects the number of images per month, with warmer colors representing cases with larger number of images. Light-gray colored tiles denote months where no imagery were available.

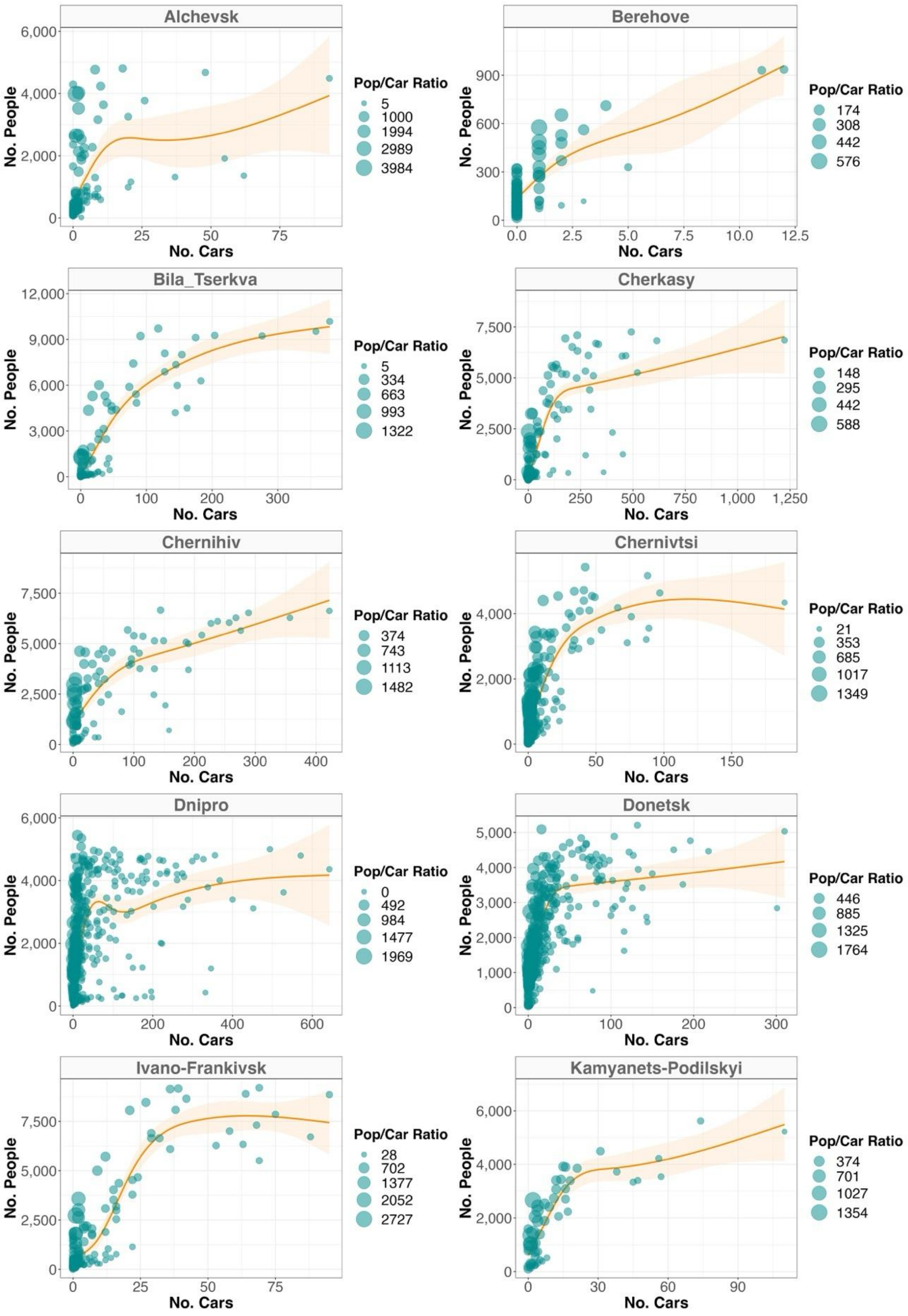

Figure S3: Relationship between the gridded average number of people and cars during the reference year (2019) for the selected Ukrainian cities. Each circle represents a unique spatial grid cell (1 x 1 km), with its size reflecting the population/car ratio. The orange smoothed function highlights the trend line from the GAM model bounded by its 95% confidence interval.

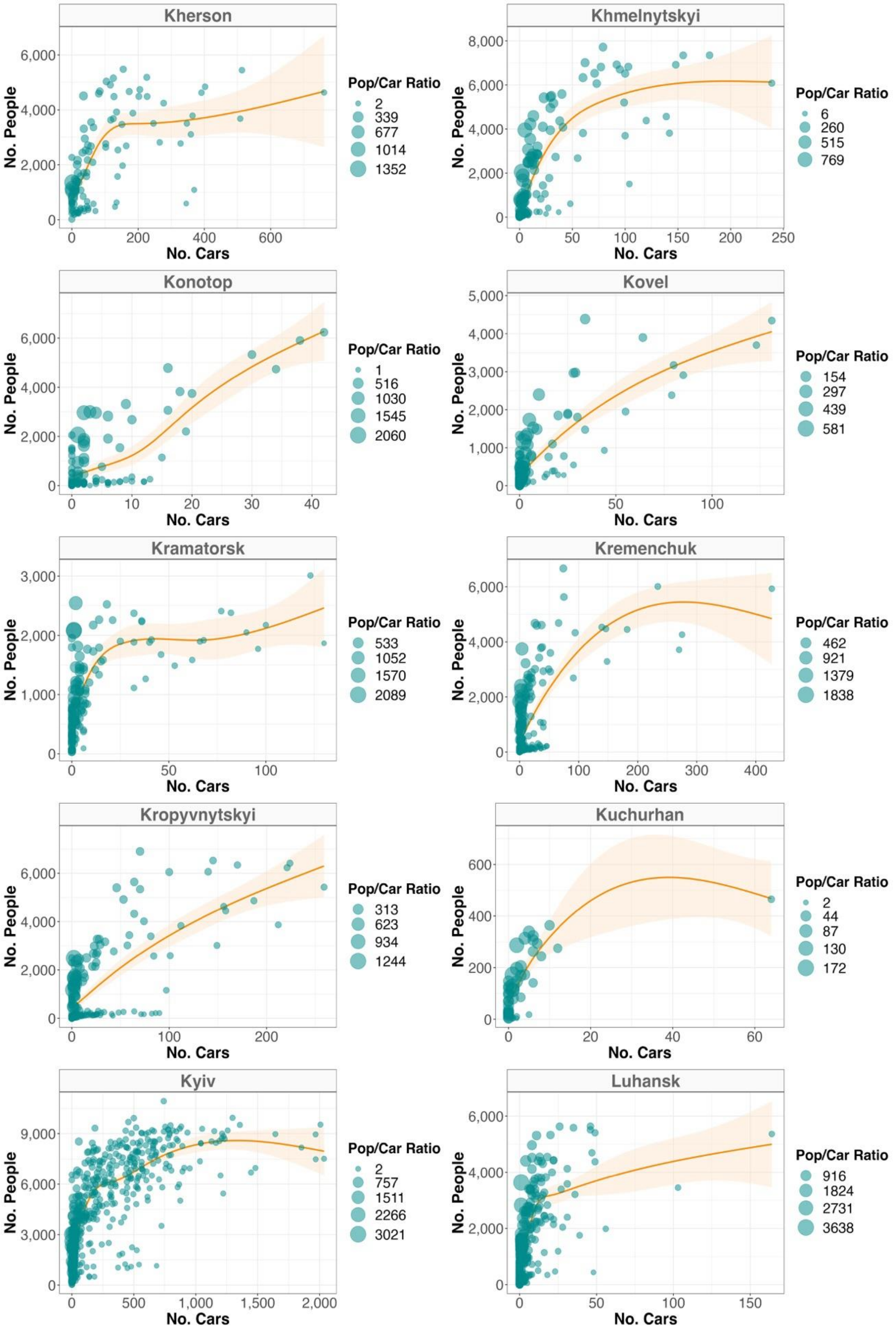

Figure S4: Cont. - Relationship between the gridded average number of people and cars during the reference year (2019) for the selected Ukrainian cities. Each circle represents a unique spatial grid cell (1 x 1 km), with its size reflecting the population/car ratio. The orange smoothed function highlights the trend line from the GAM model bounded by its 95% confidence interval.

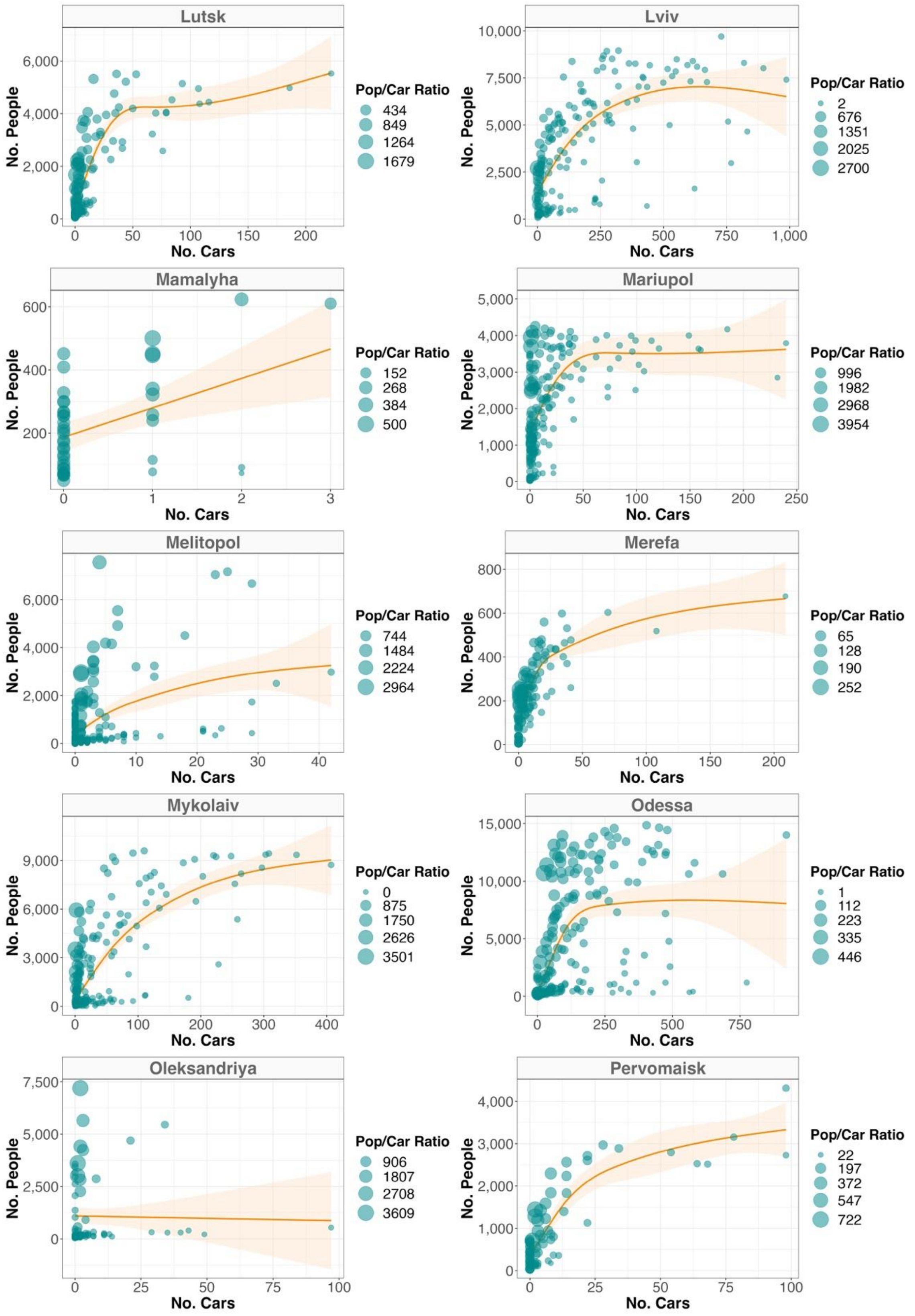

Figure S5: Cont. - Relationship between the gridded average number of people and cars during the reference year (2019) for the selected Ukrainian cities. Each circle represents a unique spatial grid cell (1 x 1 km), with its size reflecting the population/car ratio. The orange smoothed function highlights the trend line from the GAM model bounded by its 95% confidence interval.

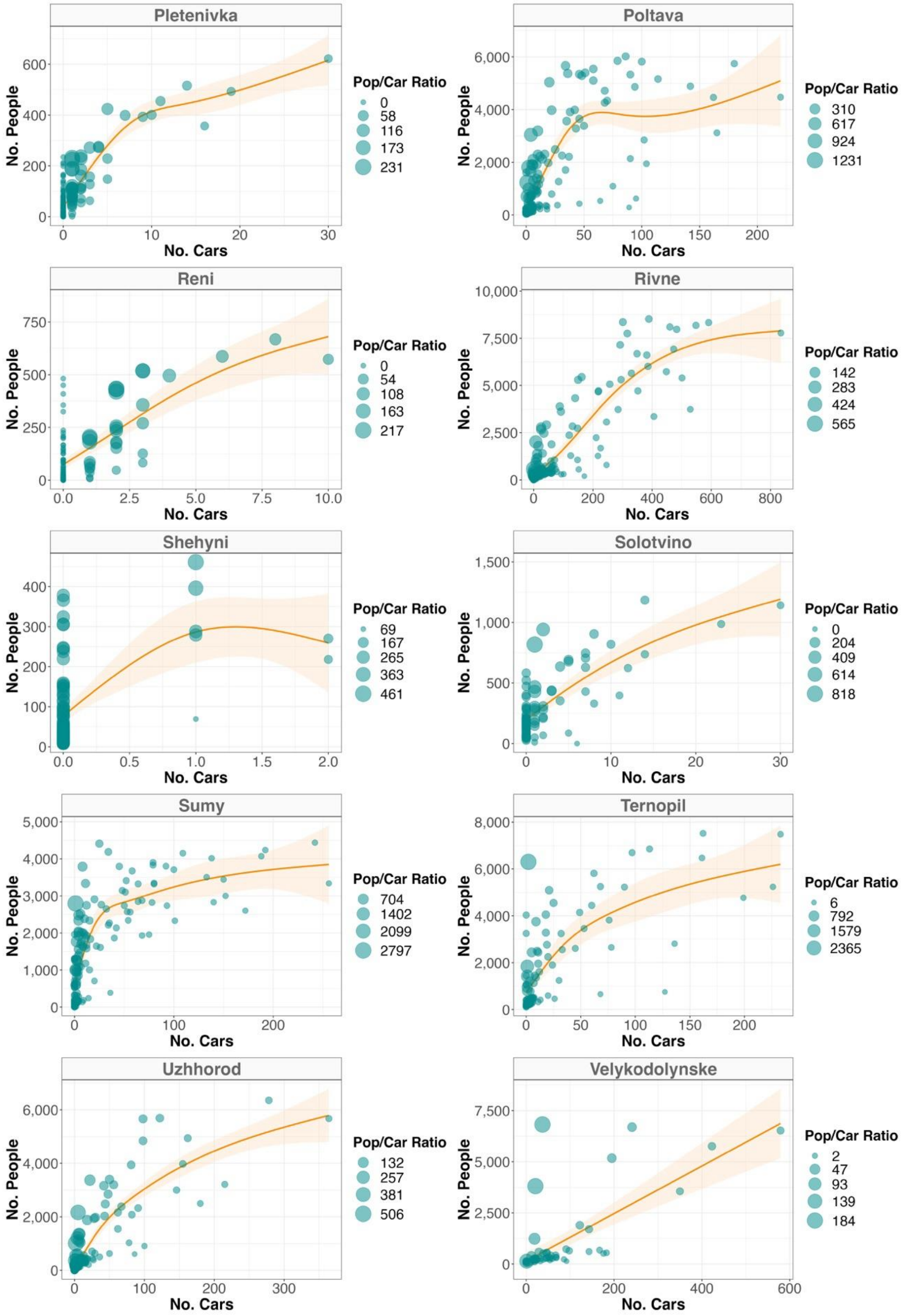

Figure S6: Cont. - Relationship between the gridded average number of people and cars during the reference year (2019) for the selected Ukrainian cities. Each circle represents a unique spatial grid cell (1 x 1 km), with its size reflecting the population/car ratio. The orange smoothed function highlights the trend line from the GAM model bounded by its 95% confidence interval.

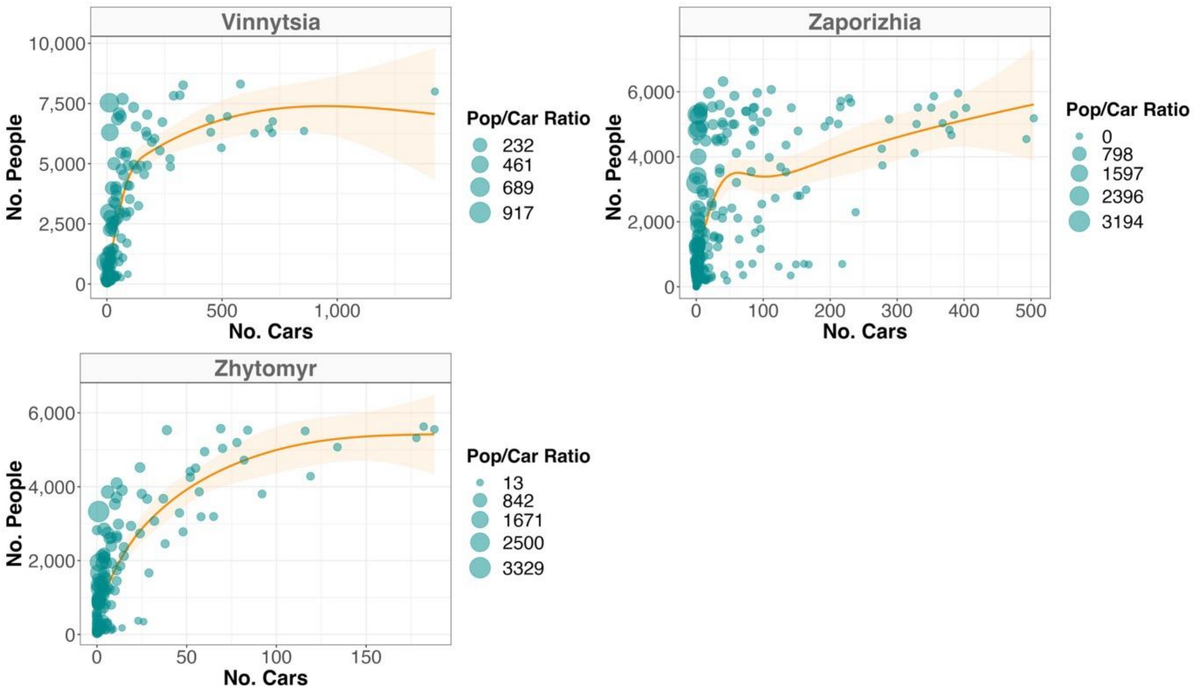

Figure S7: Cont. - Relationship between the gridded average number of people and cars during the reference year (2019) for the selected Ukrainian cities. Each circle represents a unique spatial grid cell (1 x 1 km), with its size reflecting the population/car ratio. The orange smoothed function highlights the trend line from the GAM model bounded by its 95% confidence interval.

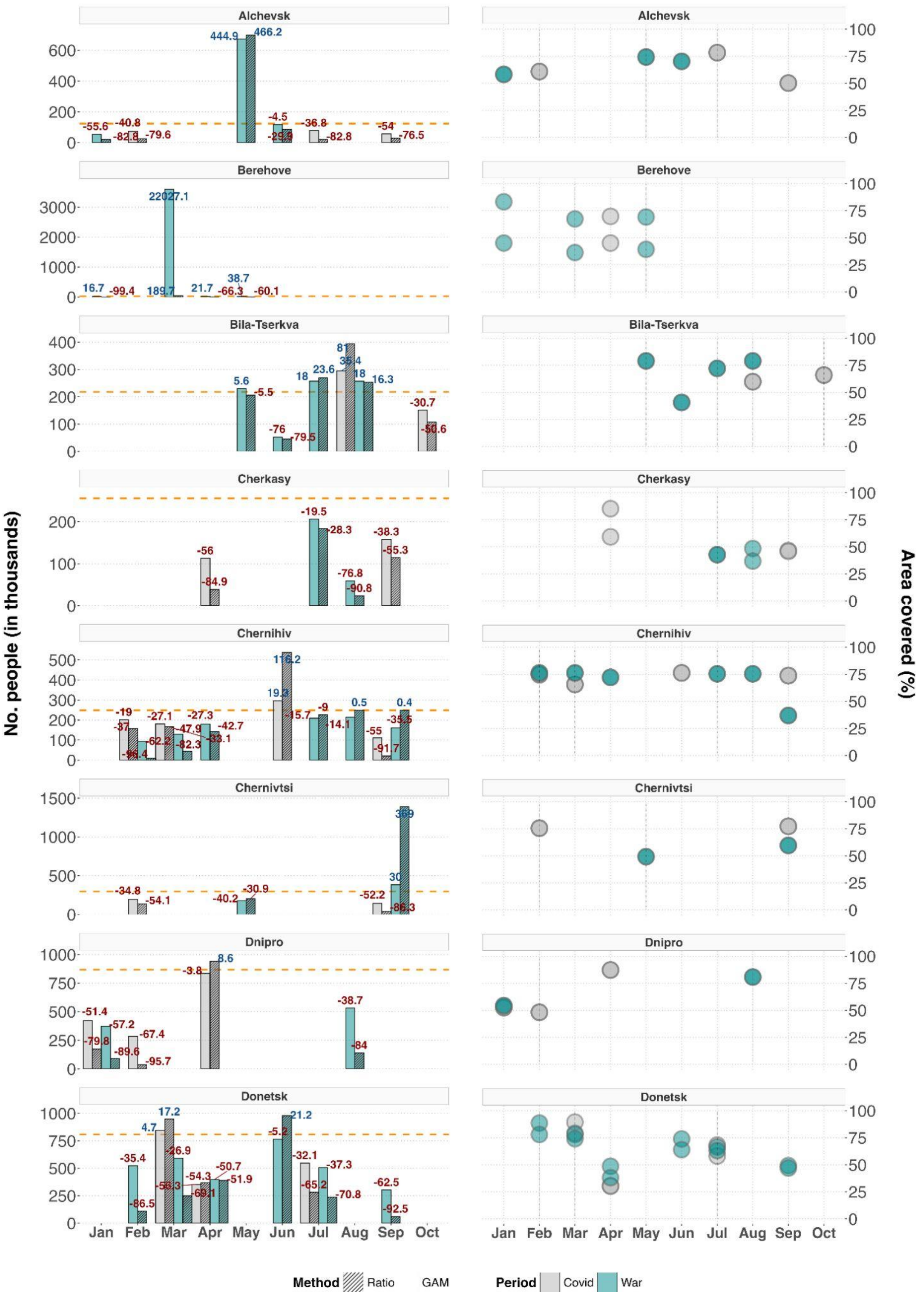

Figure S8: Predictions of internally displaced people across three different cities in Ukraine (left panels). The orange line marks the pre-War population size (2019), from which relative change in population size has been derived for the applicable months in either 2020 (first COVID-19 year, grey bars) or 2022 (War year, turquoise bars). Numbers on top of each bar denote the relative population change (in %), with colors reflecting either an increase (blue) or decrease (red). Dashed and plain bars distinguish the two tested prediction methods: linear ratio (dashed) and Generalized Additive Model (GAM, plain). Right panels depict the percentage of area covered by the satellite images underlying a given month relative to the city’s area of interest (AOI).

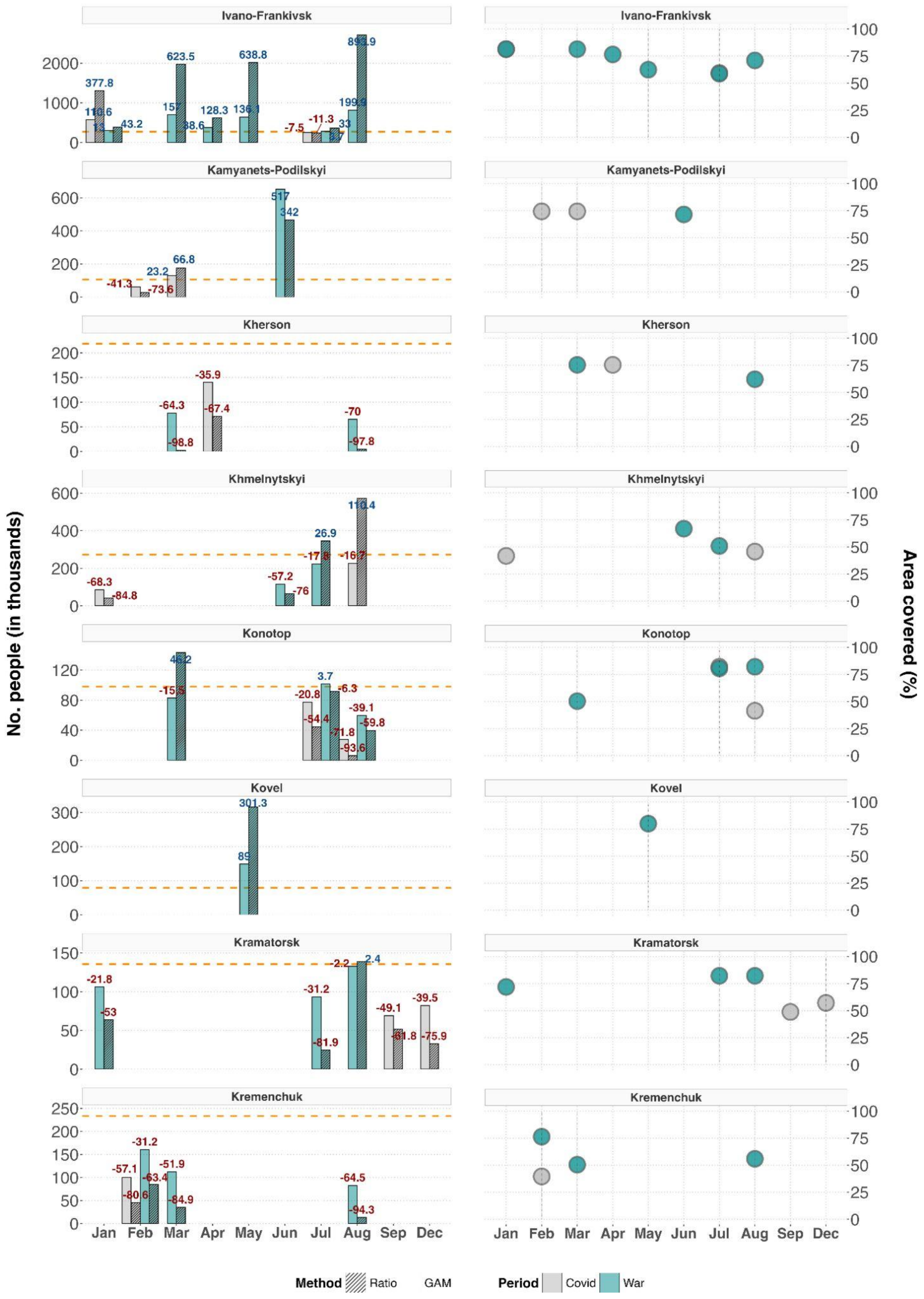

Figure S9: Cont. - Predictions of internally displaced people across three different cities in Ukraine (left panels). The orange line marks the pre-War population size (2019), from which relative change in population size has been derived for the applicable months in either 2020 (first COVID-19 year, grey bars) or 2022 (War year, turquoise bars). Numbers on top of each bar denote the relative population change (in %), with colors reflecting either an increase (blue) or decrease (red). Dashed and plain bars distinguish the two tested prediction methods: linear ratio (dashed) and Generalized Additive Model (GAM, plain). Right panels depict the percentage of area covered by the satellite images underlying a given month relative to the city's area of interest (AOI).

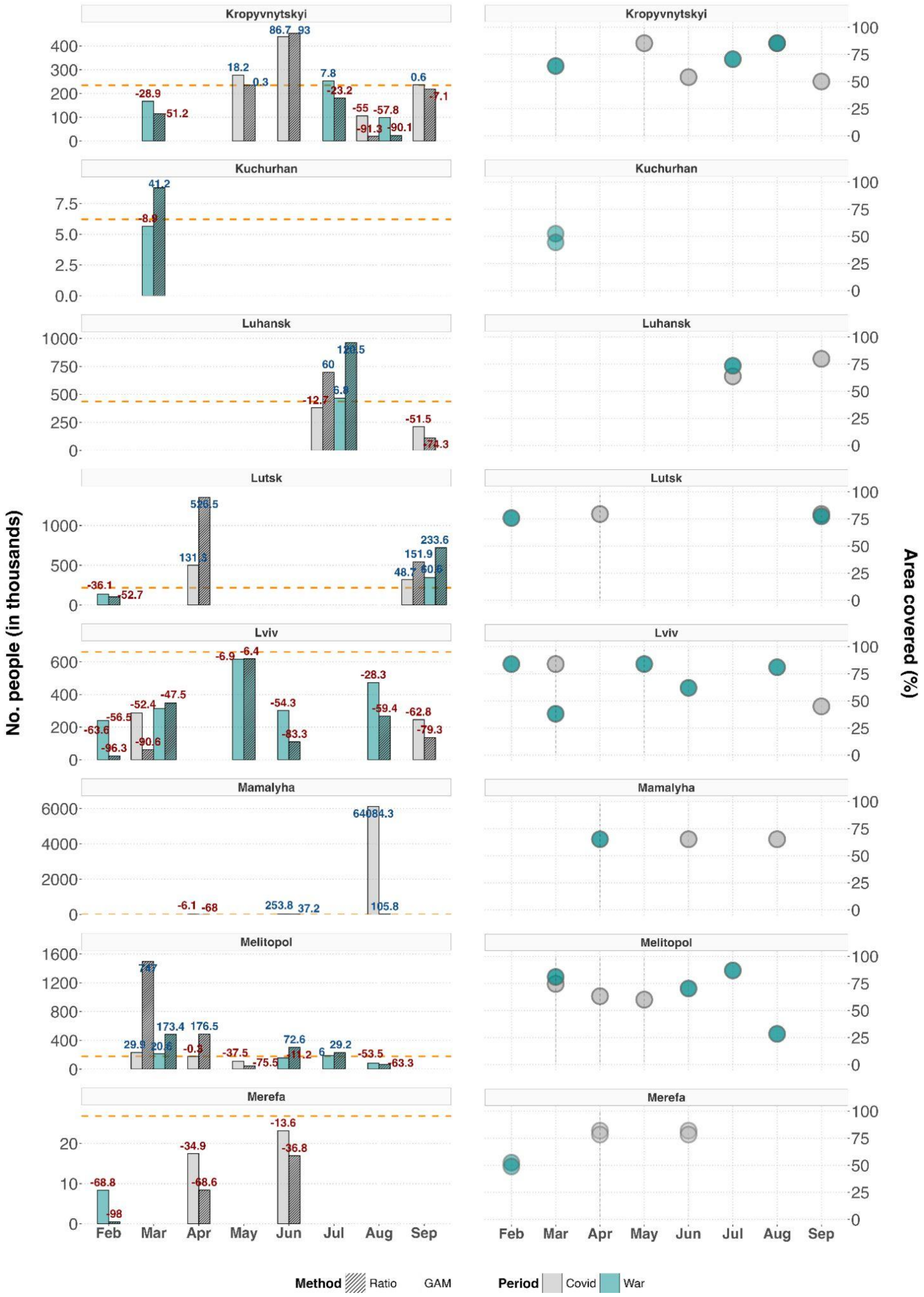

Figure S10: Cont. - Predictions of internally displaced people across three different cities in Ukraine (left panels). The orange line marks the pre-War population size (2019), from which relative change in population size has been derived for the applicable months in either 2020 (first COVID-19 year, grey bars) or 2022 (War year, turquoise bars). Numbers on top of each bar denote the relative population change (in %), with colors reflecting either an increase (blue) or decrease (red). Dashed and plain bars distinguish the two tested prediction methods: linear ratio (dashed) and Generalized Additive Model (GAM, plain). Right panels depict the percentage of area covered by the satellite images underlying a given month relative to the city's area of interest (AOI).

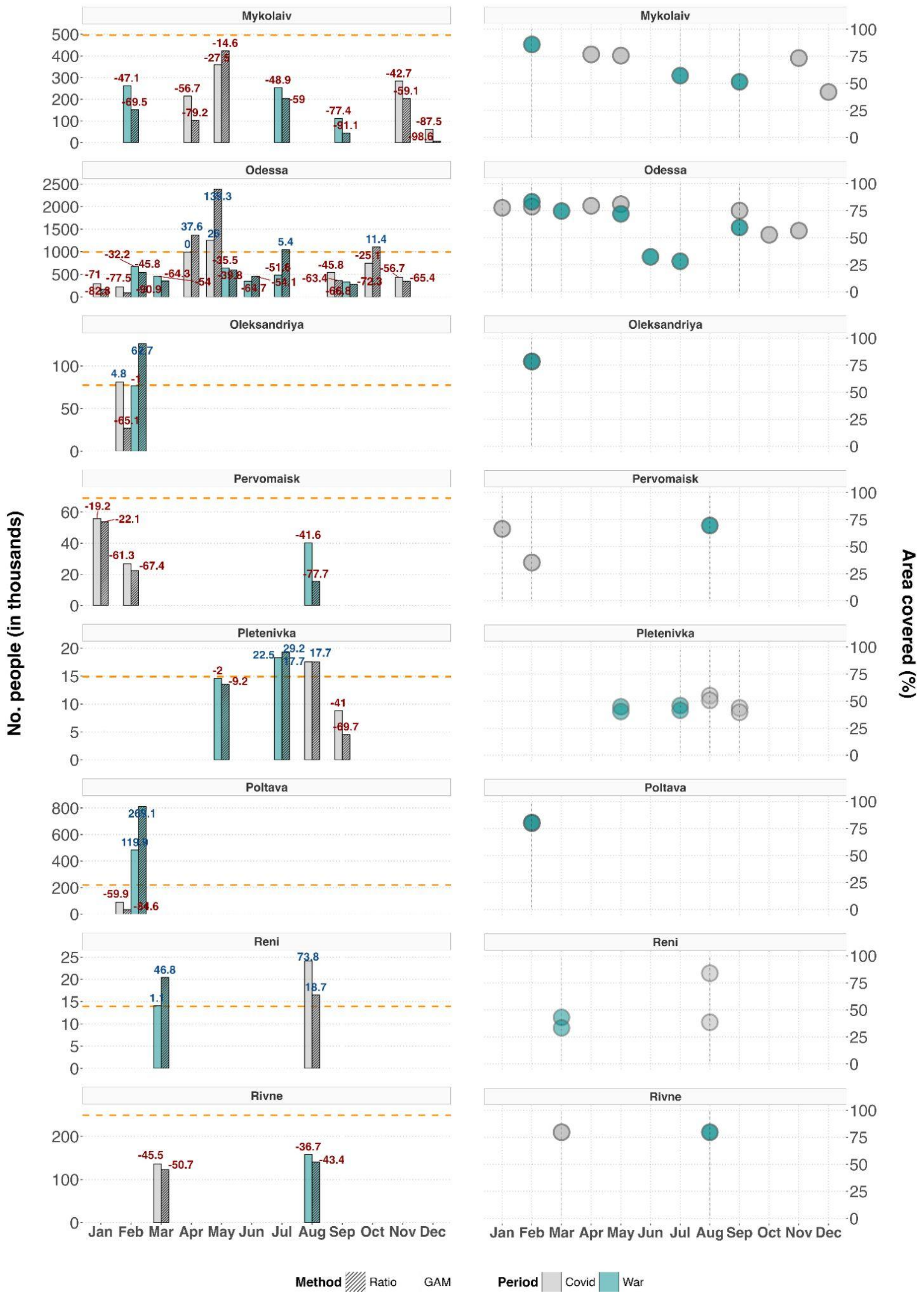

Figure S11: Cont. - Predictions of internally displaced people across three different cities in Ukraine (left panels). The orange line marks the pre-War population size (2019), from which relative change in population size has been derived for the applicable months in either 2020 (first COVID-19 year, grey bars) or 2022 (War year, turquoise bars). Numbers on top of each bar denote the relative population change (in %), with colors reflecting either an increase (blue) or decrease (red). Dashed and plain bars distinguish the two tested prediction methods: linear ratio (dashed) and Generalized Additive Model (GAM, plain). Right panels depict the percentage of area covered by the satellite images underlying a given month relative to the city's area of interest (AOI).

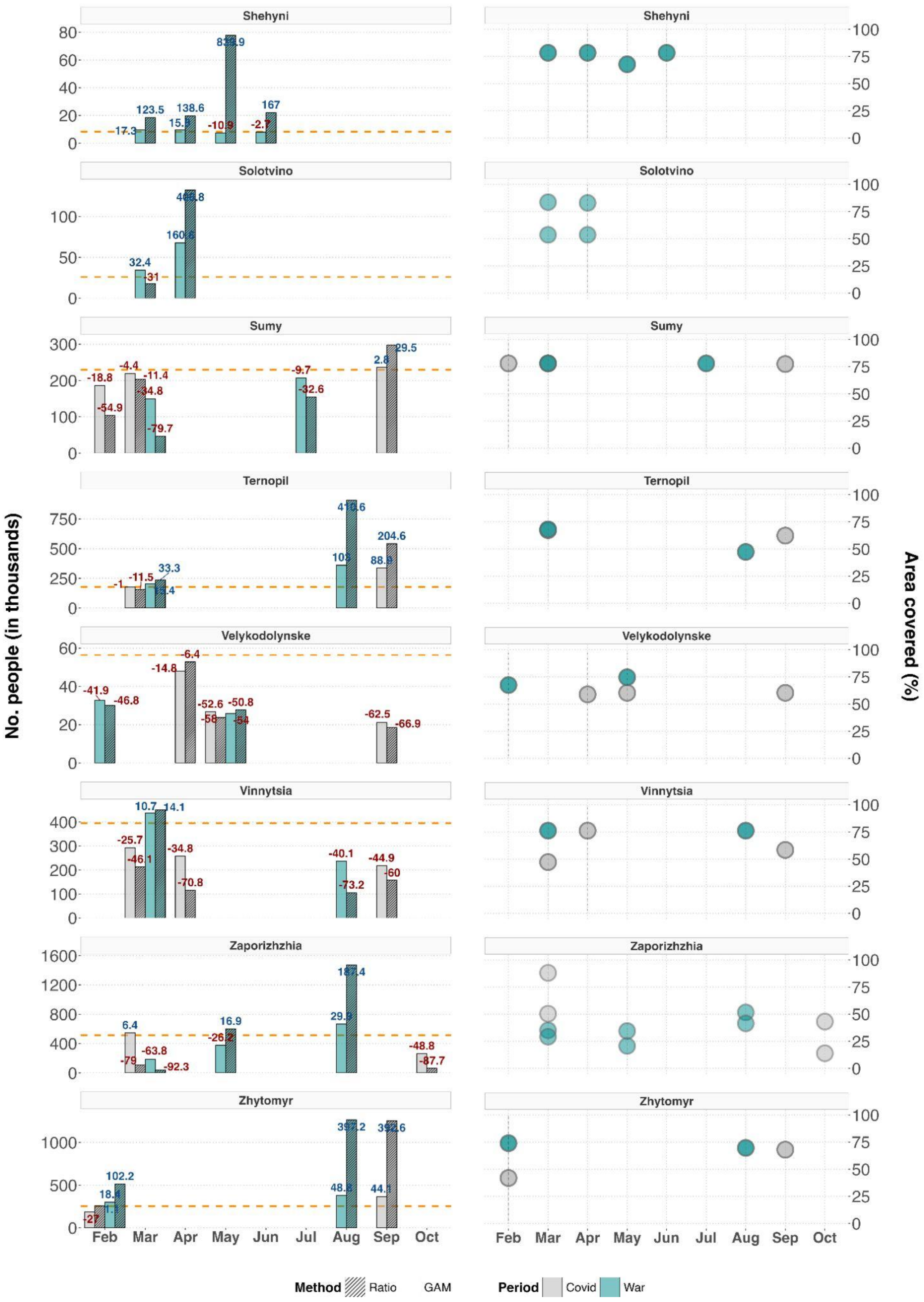

Figure S12: Cont. - Predictions of internally displaced people across three different cities in Ukraine (left panels). The orange line marks the pre-War population size (2019), from which relative change in population size has been derived for the applicable months in either 2020 (first COVID-19 year, grey bars) or 2022 (War year, turquoise bars). Numbers on top of each bar denote the relative population change (in %), with colors reflecting either an increase (blue) or decrease (red). Dashed and plain bars distinguish the two tested prediction methods: linear ratio (dashed) and Generalized Additive Model (GAM, plain). Right panels depict the percentage of area covered by the satellite images underlying a given month relative to the city's area of interest (AOI).

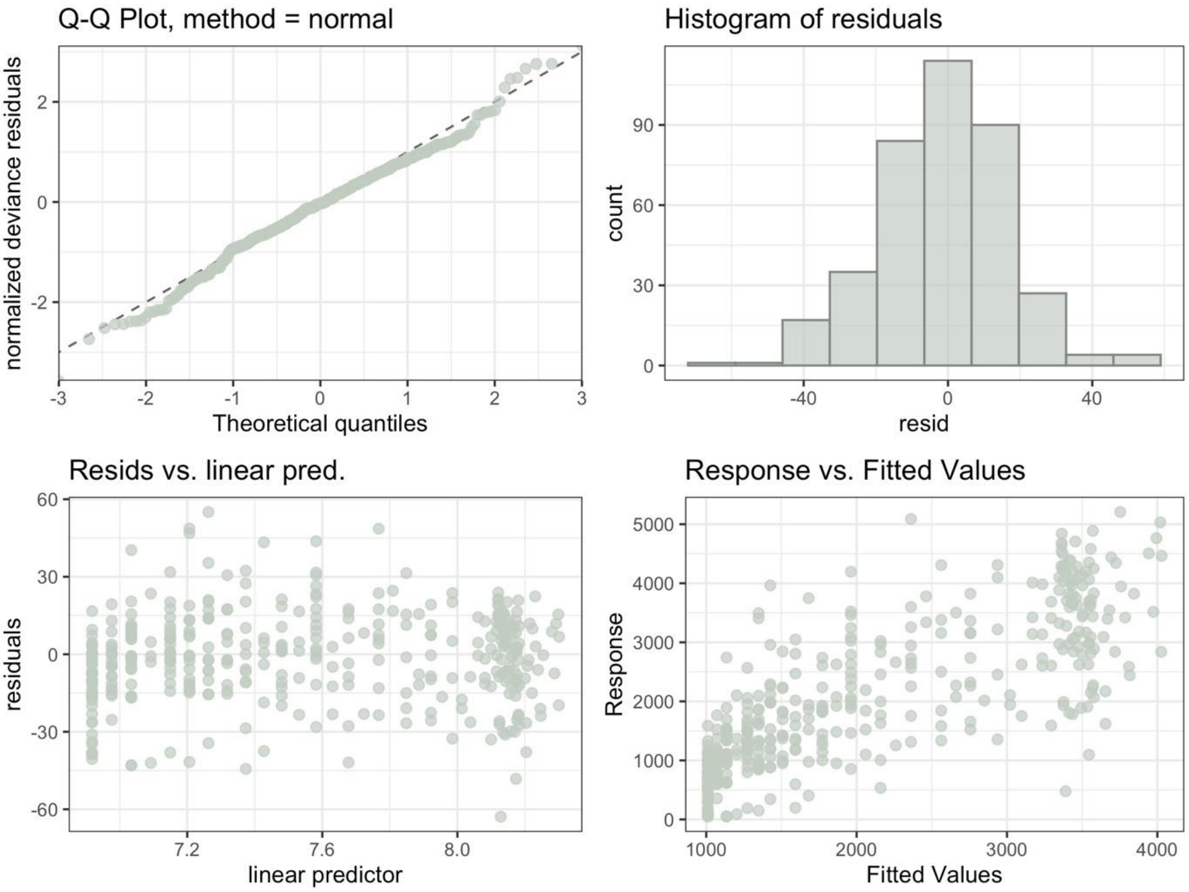

Figure S13: Visual inspection of the Generalized Additive’s Model assumption for the city of Donetsk. Upper panels show the residuals’ normality through the Q-Q plot and histogram, whereas residual’s homogeneity is confirmed in the lower left panel (no trend should be detected between residuals and the estimated values). The predictive quality of the model can be also traced in the lower right panel, where the observed values should be linearly and tightly correlated with the values estimated by the model.

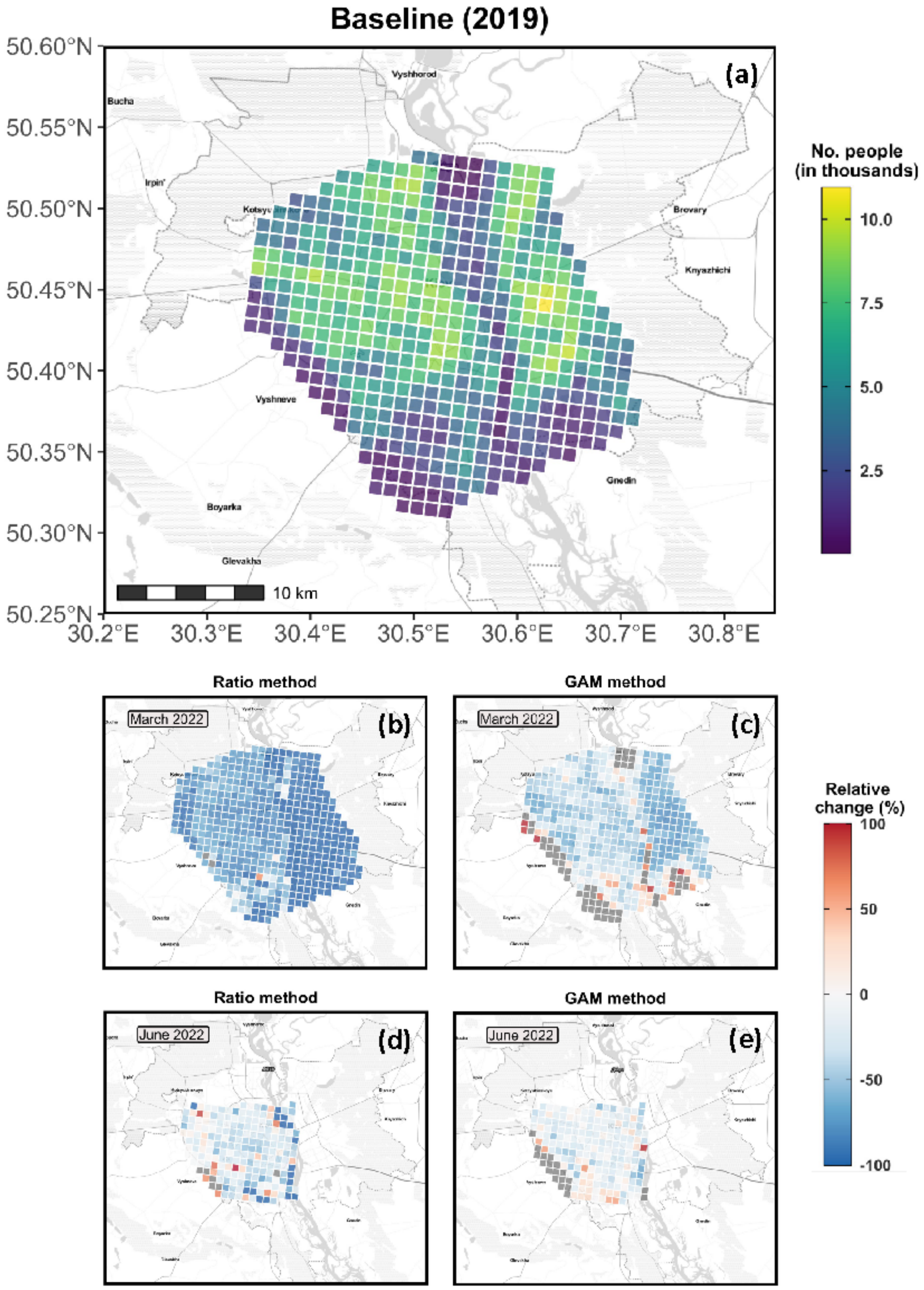

Figure S14: Gridded population for Kiev city, with each grid cell measuring 1 x 1 km. The baseline population (top panel) was retrieved from WorldPop's database, whereas the population for March and June 2022 were predicted through either the Ratio (left mid and lower panels) or the Generalized Additive Model (GAM, right mid and lower panels) method. Color scale in the four lower panels reflect the percentage change of the population in 2022 relative to 2019. Gray colored grid cell represent cases where the predicted population exceeded the limits of the color range, and were thus omitted to maintain a discernible color scale.

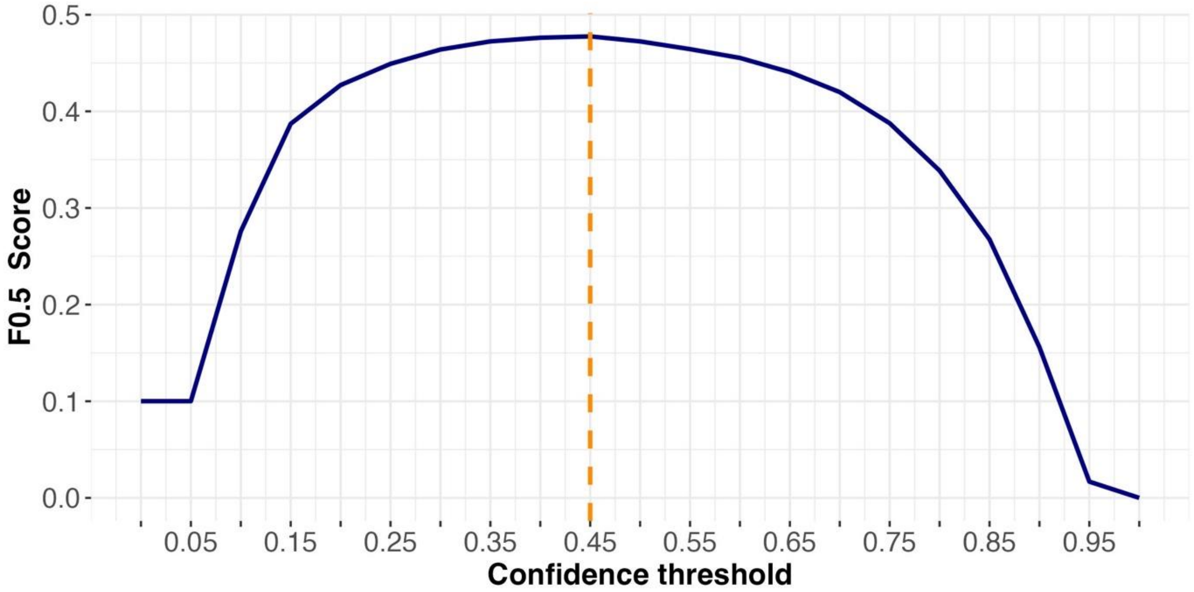

Figure S15: Results from the sensitivity test to evaluate the optimal confidence threshold to detect cars from the satellite images. A F-beta score of 0.5 gives more weight to precision than recall, minimising as such the detection of false-positives.

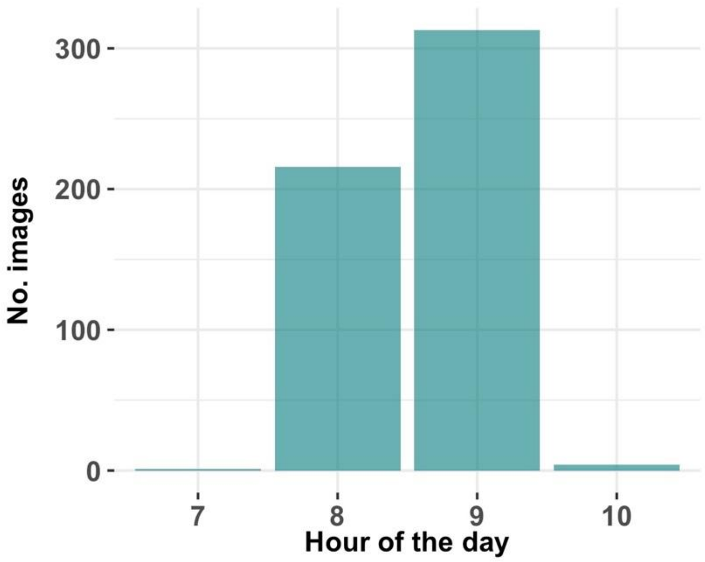

Figure S16: Number of satellite images per hour of the day (UTC time zone).

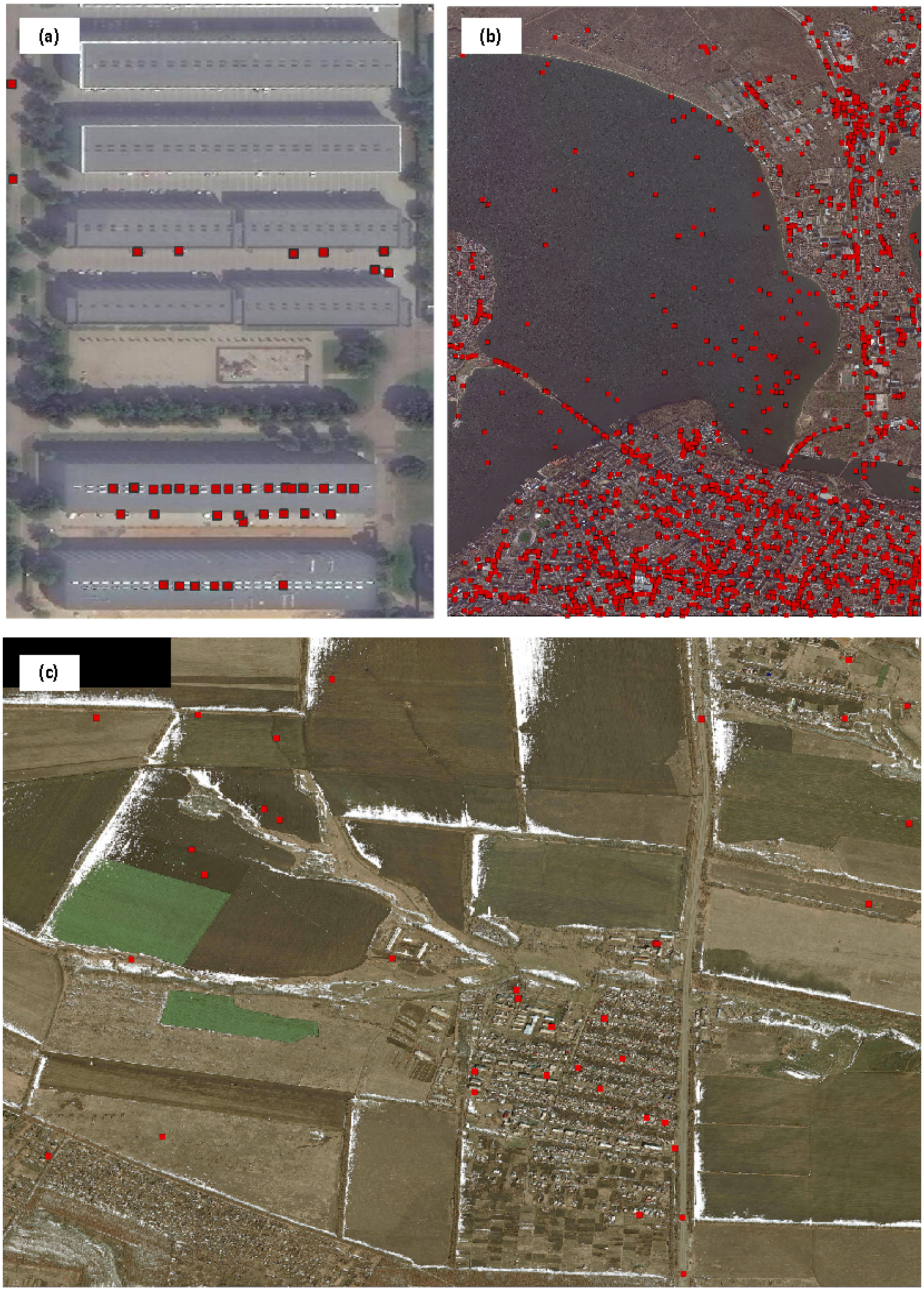

Figure S17: Satellite images showing false car detections marked by the red squares. The three panels illustrate three distinct types of false-positives: (a) on air-conditioners fixed on top of commercial buildings in Odessa, b) on the Pivdennyi Buh River in Mykolaiv, and (c) on crop fields in Luhansk. Satellite images © 2019–2023 Maxar Technologies

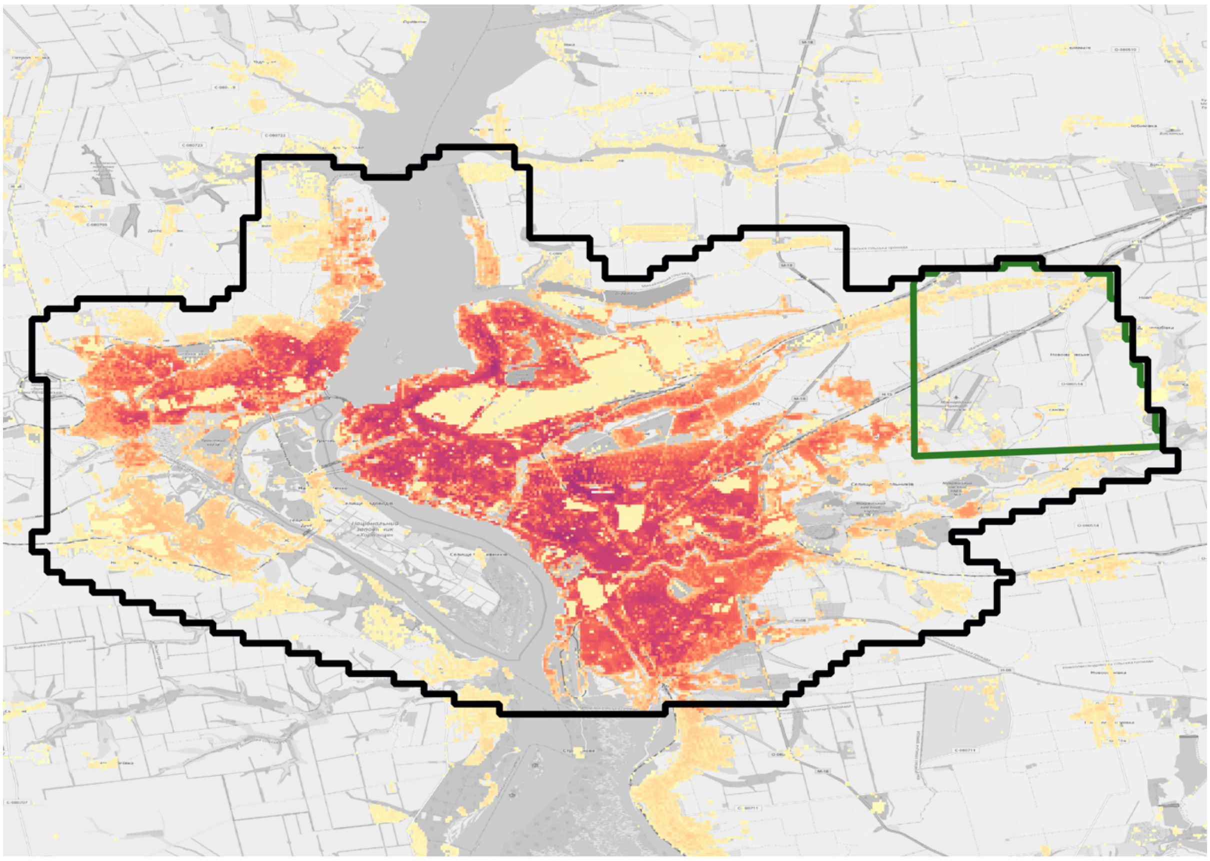

Figure S18: Population distribution for the city of Zaporizhzhia overlaid to the spatial extent of two different satellite images. The black and green line refers to the images taken in 19.03.2020 and 23.01.2020, respectively. While the first image covers the full extent of the Area of Interest (AOI), hence 100% of the population distribution, the second image covers less than 50% of the AOI which is located on an area that represents less than 1% of the city's population.

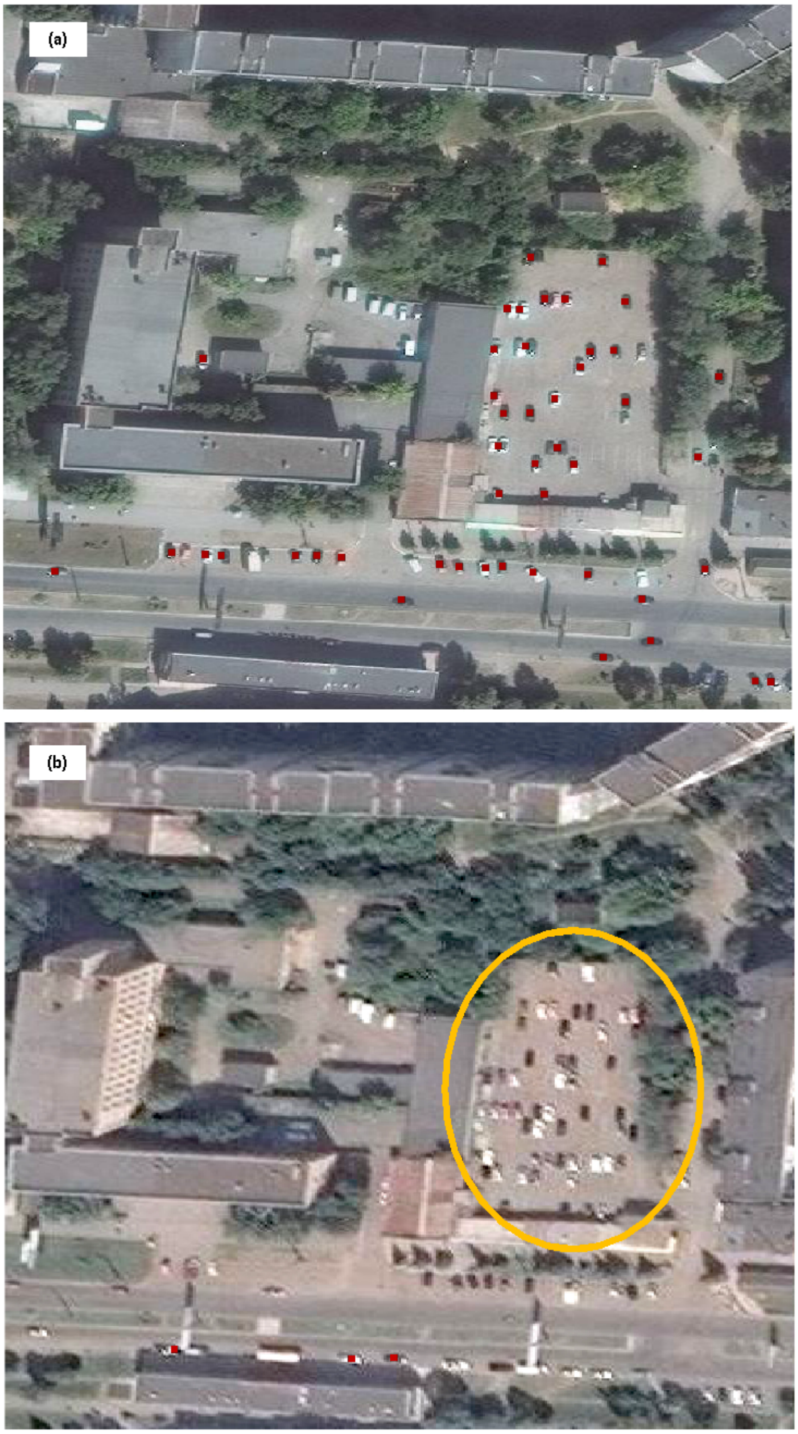

Figure S19: Illustrative example from Alchevsk city on the effect of image resolution on car detection (marked as red squares in the images). The two panels shows a parking lot in front of a post office, whereby the Convolutional Neural Network (CNN) model detects visibly more cars in the higher- resolution image (0.3 m GSD; panel (a)) than in the lower-resolution image (0.5 m GSD; panel (b)). Note that both images were taken on similar time and dates (a = 12.09.2019 08:55 UTC, b = 24.08.2019 08:22 UTC), as well as had similar imagery features: off-Nadir angle (a = 17.56°, b = 24.14°), sun elevation (a = 45.58°, b = 50.42°). The orange circle in panel (b) highlights the area where cars remained largely undetected. Satellite images © 2019–2023 Maxar Technologies.

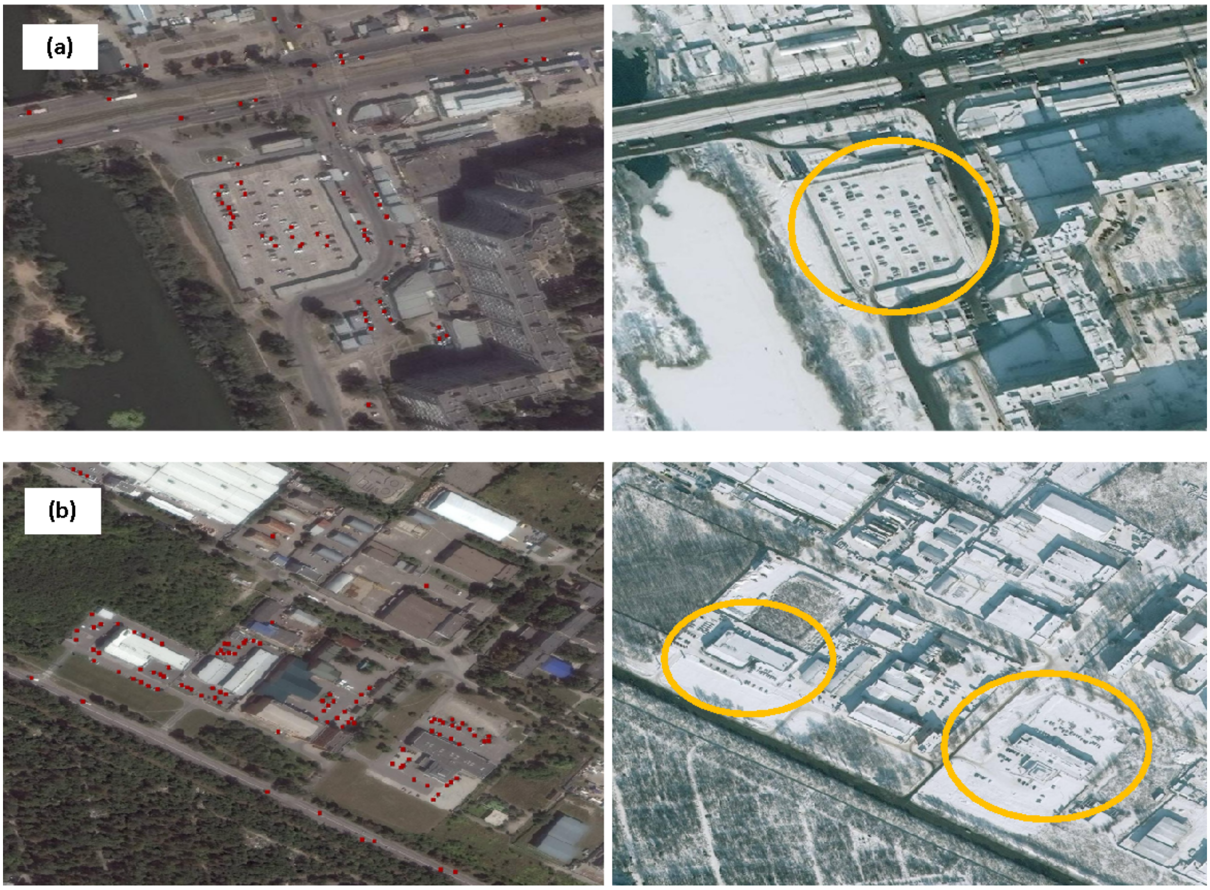

Figure S20: Illustrative example from Dnipro city to show the effect of snow on the detectability of cars. Both panels highlight two distinct commercial areas (a, b), with left and right panels showing the detected cars (red squares) on a summer and winter day, respectively. In the presence of snow (right panels), cars covered by snow remained undetected due to the reduced contrast. The orange circles in the right-side panels highlight the areas where cars remained largely undetected. Satellite images © 2019–2023 Maxar Technologies.

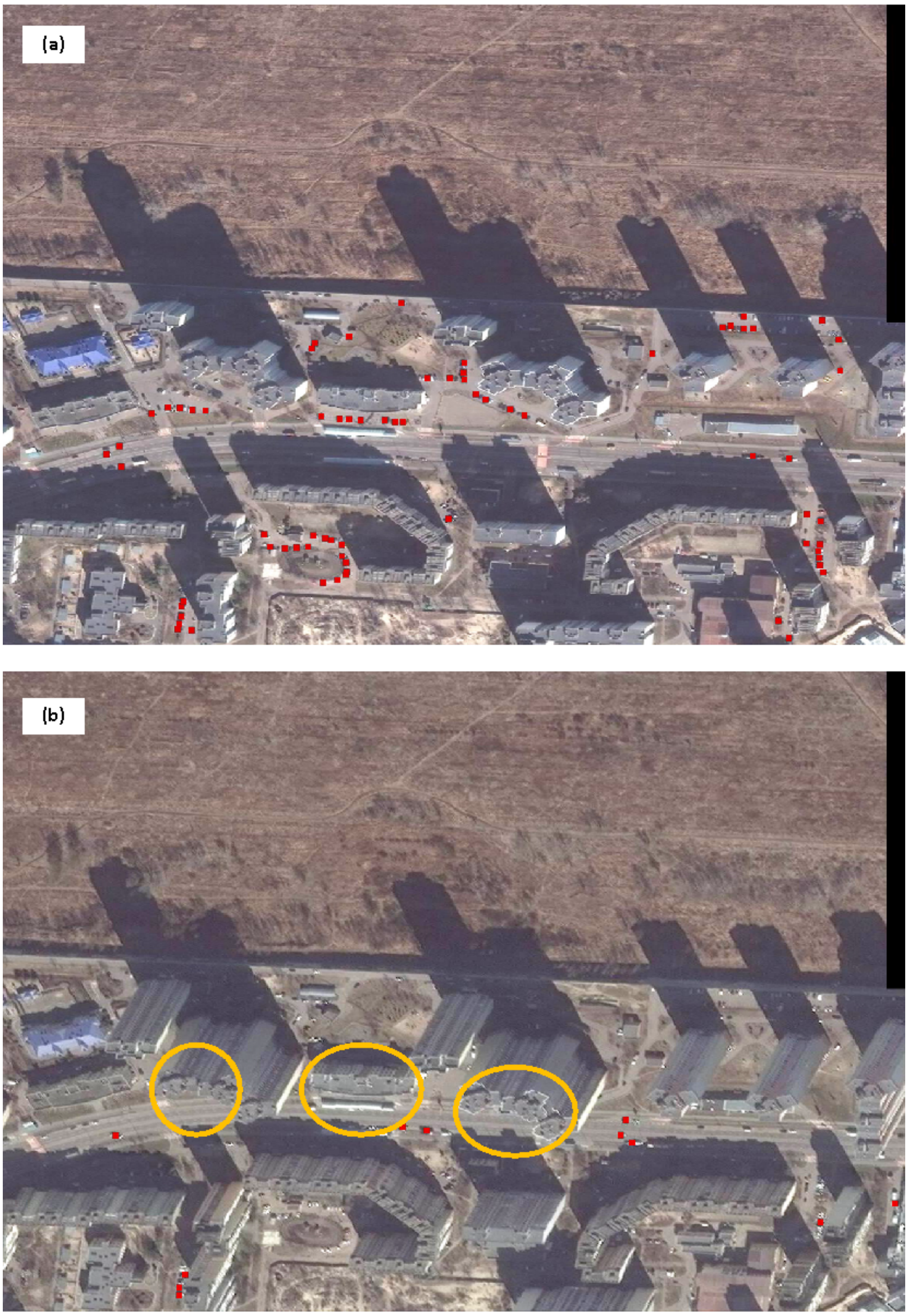

Figure S21: Snapshots from the same residential area in Kiev city to illustrate the off-Nadir angle effect on the detectability of cars (a = 10.84°, b = 37.08°). The two panels clearly show that more cars are detected at smaller off-Nadir angles (a). At larger viewing angles (b), the buildings tend to occlude other important infrastructure such as roads and parking lots and obfuscate the circulating cars, thus reducing the detectability. Note that both images were taken on similar time of the day (a = 09:06 UTC, b = 09:05 UTC), while also had similar imagery features: sun elevation angle (a = 30.17°, b = 37.58°), image resolution (a = b = 0.5 m GSD). The orange circles in the lower panel highlights the areas where cars remained largely undetected. Satellite images © 2019–2023 Maxar Technologies.

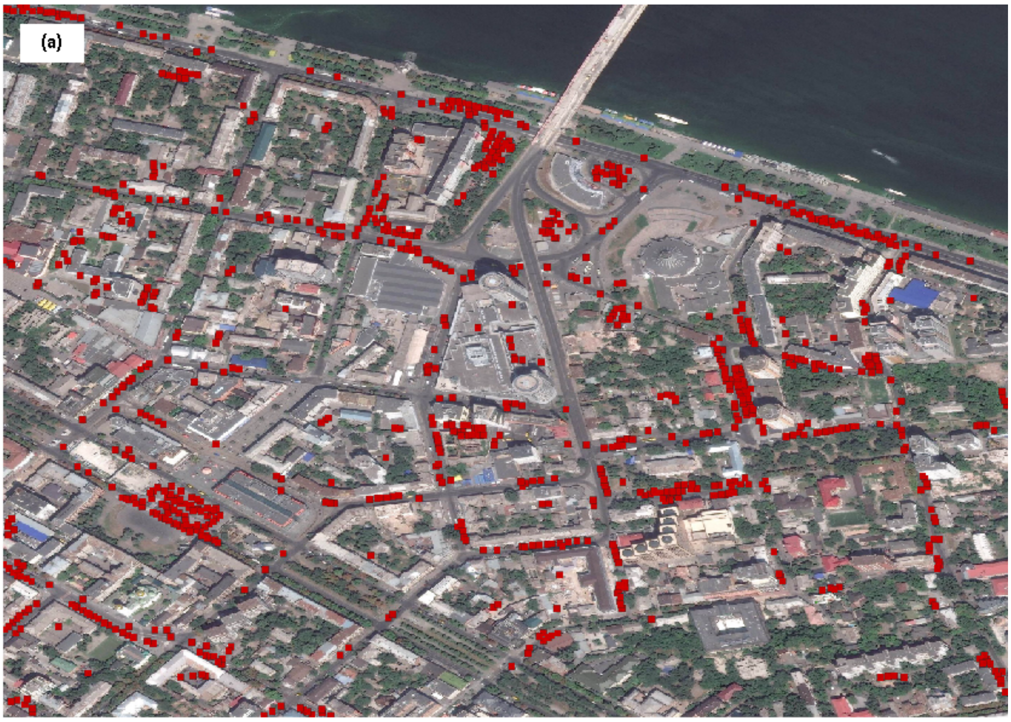

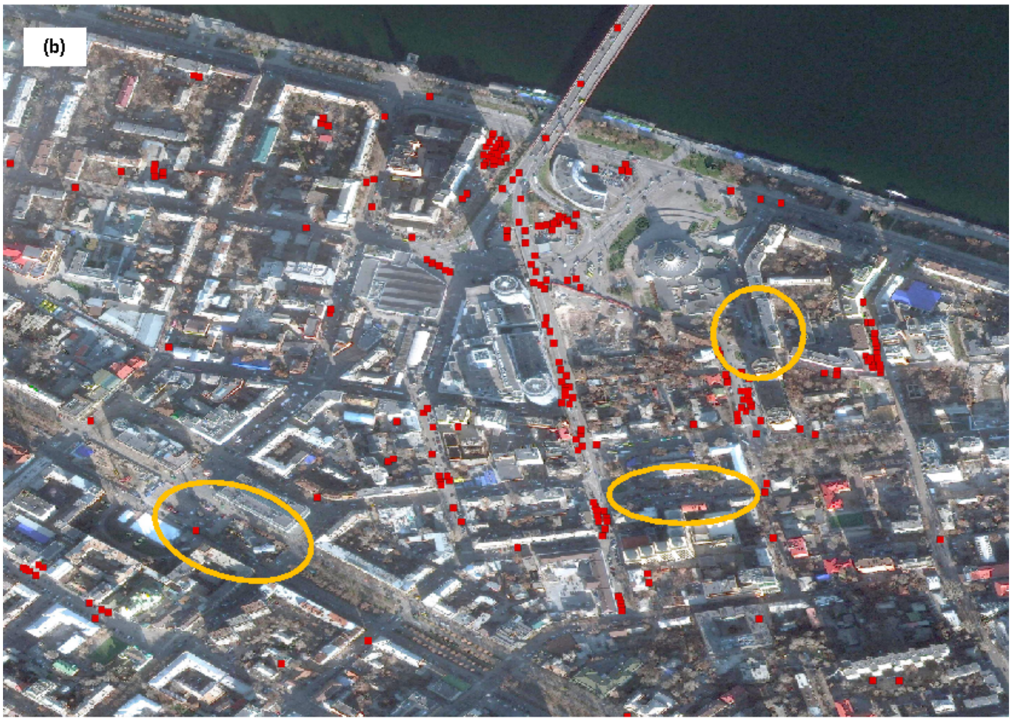

Figure S22: Snapshots from the same commercial area in Dnipro city to illustrate the effect of sun elevation angle on the detectability of cars (a = 58.68°, b = 17.96°). At greater angles, the shadows created by the buildings are reduced, resulting in greater discernability of the circulating cars as in Panel (a) whereas Panel (b) shows that fewer cars are detected at smaller angles. Note that both images were taken on similar time of the day (a = 08:36 UTC, b = 08:41 UTC), while also had similar imagery features: off-Nadir angle (a = 17.81°, b = 12.69°), image resolution (a = b = 0.4 m GSD). The orange circles in the lower panel highlights the areas where cars remained largely undetected. Satellite images © 2019–2023 Maxar Technologies.

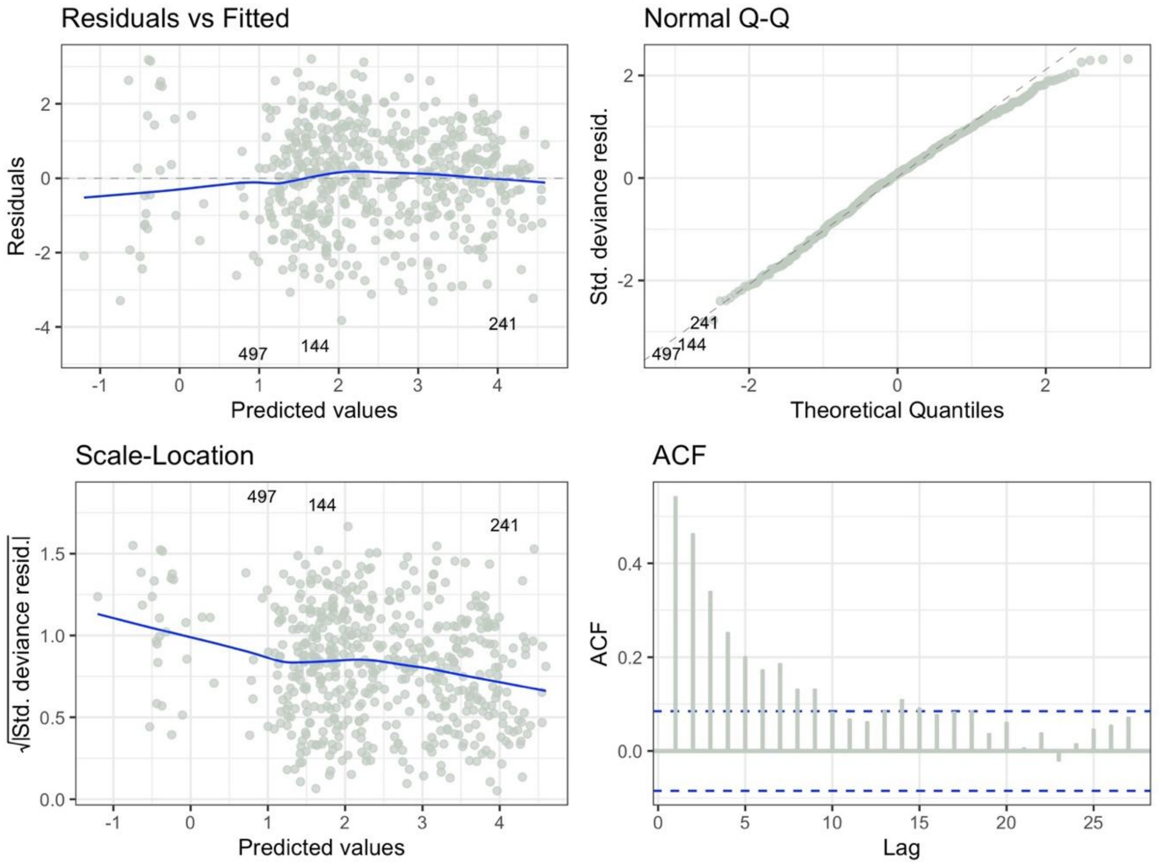

Figure S23: Visual diagnostics of the Generalized Linear Model (GLM) applied to evaluate the effect of satellite imagery-related covariates on car detection.

Supplementary Tables

Table S1: Goodness-of-fit statistics of the Generalized Additive Models (GAMs) applied to the baseline year.

City	R^2(adj.)
Alchevsk	0.30
Berehove	0.63
Bila-Tserkva	0.83
Cherkasy	0.74
Chernihiv	0.62
Chernivtsi	0.65
Dnipro	0.35
Donetsk	0.60
Ivano-Frankivsk	0.83
Kamyanets-Podilskyi	0.75
Kherson	0.50
Khmelnytskyi	0.67
Konotop	0.60
Kovel	0.65
Kramatorsk	0.54
Kremenchuk	0.47
Kropyvnytskyi	0.54
Kuchurhan	0.61
Kyiv	0.63
Luhansk	0.62
Lutsk	0.71
Lviv	0.52
Mamalyha	0.21
Mariupol	0.37
Melitopol	0.23
Merefa	0.66
Mykolaiv	0.61
Odessa	0.35
Oleksandriya	-0.04
Pervomaisk	0.75
Pletenivka	0.74
Poltava	0.61
Reni	0.50
Rivne	0.79
Shehyni	0.26
Solotvino	0.53
Sumy	0.63
Ternopil	0.55
Uzhhorod	0.75
Velykodolynske	0.47
Vinnytsia	0.67
Zaporizhia	0.45
Zhytomyr	0.68

Table S2: Summary of results achieved by the car detection model in terms of Precision, Recall, and $F_{0.5}$-Score across varying confidence thresholds.

Confidence	Precision	Recall	$F_{0.5}$-Score
0.00	0.0821	0.8199	0.1001
0.05	0.0821	0.8199	0.1001
0.10	0.2408	0.6605	0.2758
0.15	0.3614	0.5413	0.3871
0.20	0.4165	0.4760	0.4272
0.25	0.4542	0.4302	0.4492
0.30	0.4858	0.3933	0.4640
0.35	0.5119	0.3611	0.4724
0.40	0.5343	0.3318	0.4762
**0.45**	**0.5578**	**0.3032**	**0.4776**
0.50	0.5745	0.2761	0.4724
0.55	0.5926	0.2489	0.4644
0.60	0.6117	0.2252	0.4553
0.65	0.6319	0.1992	0.4406
0.70	0.6563	0.1720	0.4199
0.75	0.6807	0.1424	0.3877
0.80	0.7045	0.1100	0.3386
0.85	0.7390	0.0753	0.2675
0.90	0.7556	0.0374	0.1560
0.95	0.8228	0.0034	0.0168
1.00	0.0000	0.0000	0.0000

Table S3: List of the selected cities with descriptive statistics relative to their satellite imagery coverage. The AOI defines roughly the city's perimeter, whereby the downloaded satellite images might or not cover the full AOI area. On average, most cities were well covered by the downloaded images. Berdychiv was the only city with only one available image, thus the NA in the table.

City	Oblast	Region	AOI area (km2)	Mean	Std. Dev.
Alchevsk	Luhansk	East	161.5	69.7	53.1
Berdychiv	Zhytomyr	Central	62.8	62.8	NA
Berehove	Zakarpattia	West	184.3	90.4	59
Bila-Tserkva	Kiev	Central	142.7	113.2	40.2
Cherkasy	Cherkasy	Central	444.3	206.2	126.3
Chernihiv	Chernihiv	Central	128.3	125.8	37.3
Chernivtsi	Chernivtsi	West	310.5	282.3	81.7
Chortkiv	Ternopil	West	64.8	24.4	20.9
Dnipro	Dnipropetrovsk	East	525.5	273.3	169.7
Donetsk	Donetsk	East	509.3	149.2	146.9
Drohobych	Lviv	West	230.4	209.4	30.6
Hremiach	Chernihiv	Central	34.7	16.5	11.9
Ivano-Frankivsk	Ivano-Frankivsk	West	178.7	132.6	40.7
Kamyanets-Podilskyi	Khmelnytskyy	West	74.5	74.5	1.1
Katerynivka	Sumy	Central	364	207.6	92.1
Kharkiv	Kharkiv	East	418.1	215.9	115.9
Kherson	Kherson	Central	142.8	81.9	47.9
Khmelnytskyi	Khmelnytskyy	West	188.1	101.4	66
Kolomyiska	Ivano-Frankivsk	West	324.9	220.1	93.6
Konotop	Sumy	Central	145.4	145.4	49.7
Kovel	Volyn	West	141.4	125.4	35.2
Kozyatyn	Vinnytsia	Central	139.1	75.3	90.2
Kramatorsk	Donetsk	East	167.7	104.5	53.6
Kremenchuk	Poltava	Central	332.6	164.2	117.2
Kropyvnytskyi	Kirovohrad	Central	240.2	165.8	67.3
KryvyiRih	Dnipropetrovsk	Central	643.2	290.5	214.7
Kuchurhan	Odessa	Central	71.9	61.5	22
Kyiv	Kiev	Central	569.7	201.1	186
Luhansk	Luhansk	East	475.9	72.9	153.8
Lutsk	Volyn	West	207.5	186.9	57.7
Lviv	Lviv	West	217	109.7	76.3
Mamalyha	Chernivtsi	West	70.1	51.2	27
Mariupol	Donetsk	East	257.7	164.2	89.8
Melitopol	Zaporizhzhya	East	298.9	227.7	84.1
Merefa	Kharkiv	East	153	64.9	63.2
Milove	Luhansk	East	73.2	40.3	24

Mykolaiv	Mykolayiv	Central	374.7	131.6	113.6
Nizhyn	Chernihiv	Central	101.9	99	29.3
Odessa	Odessa	Central	292	194.4	78.7
Oleksandriya	Kirovohrad	Central	97	97	36.2
Pervomaisk	Mykolayiv	Central	103.2	53.3	37.3
Pletenivka	Kharkiv	East	255.1	100.8	81.4
Poltava	Poltava	East	190.2	187.5	51.3
Porubne	Chernivtsi	West	225.8	122.1	75.3
Rava-Ruska	Lviv	West	147.7	83.6	51.7
Reni	Odessa	Central	288.7	89.5	79.2
Rivne	Rivne	West	219.7	210.8	84.7
Sarny	Rivne	West	146.4	146.4	0.5
Shehyni	Lviv	West	124.1	83.6	42.4
Smila	Cherkasy	Central	361.1	208.4	78.9
Solotvino	Zakarpattia	West	163.4	108.3	59.5
Storozhynets	Chernivtsi	West	104.9	39.8	31.1
Sumy	Sumy	East	195.7	182.6	75.4
Ternopil	Ternopil	West	149.6	114	28.9
Udobne	Odessa	Central	198.3	107.2	58.4
Uzhhorod	Zakarpattia	West	263.4	145.3	77.8
Velyki-Kopani	Kherson	Central	96.3	40.9	37.5
Velykodolynske	Odessa	Central	74.5	48	27.8
Vinnytsia	Vinnytsia	Central	225.1	198.4	75.9
Zaporizhzhia	Zaporizhzhya	East	499.6	204.8	172.4
Zhytomyr	Zhytomyr	Central	198.7	105.1	71.3

Table S4: Summary of the Open Street Maps (OSM) tags retrieved for the false-positives filtering process. For a full description of each OSM tag, refer to https://wiki.openstreetmap. org/wiki/Map features

Key	Value
Landuse	Farmland
	Farmyard
	Vineyard
	Forest
	Grass
	Scrub
	Meadow
	Heath
	Railway station
	Railway
	Industrial
Place	Sea
Natural	Grassland
	Water
	Wetland
	Scrub
	Wood
	Bay
Leisure	Park
Aeroway	Aerodrome
	Apron
